# Vaginal *Lactobacillus crispatus* persistence following application of a live biotherapeutic product: colonization phenotypes and genital immune impact

**DOI:** 10.1186/s40168-024-01828-7

**Published:** 2024-06-21

**Authors:** Eric Armstrong, Anke Hemmerling, Steve Miller, Sanja Huibner, Maria Kulikova, Emily Crawford, Gloria R. Castañeda, Bryan Coburn, Craig R. Cohen, Rupert Kaul

**Affiliations:** 1 Department of Medicine, University of Toronto, Toronto, Canada; 2 Department of Obstetrics, Gynecology & Reproductive Sciences, University of California, San Francisco, San Francisco, USA; 3 Department of Laboratory Medicine, University of California, San Francisco, San Francisco, USA; 4 Toronto General Hospital Research Institute, University Health Network, Toronto, Canada; 5 Department of Microbiology and Immunology, University of California, San Francisco, San Francisco, USA; 6 Chan Zuckerberg Biohub, San Francisco, USA; 7 Department of Medicine, University Health Network, Toronto, Canada

## Abstract

**Background:**

Bacterial vaginosis (BV) increases HIV acquisition risk, potentially by eliciting genital inflammation. After BV treatment, the vaginal administration of LACTIN-V, a live biotherapeutic containing the *Lactobacillus crispatus* strain CTV-05, reduced BV recurrence and vaginal inflammation; however, 3 months after product cessation, CTV-05 colonization was only sustained in 48% of participants.

**Results:**

This nested sub-study in 32 participants receiving LACTIN-V finds that 72% (23/32) demonstrate clinically relevant colonization (CTV-05 absolute abundance > 10^6^ CFU/mL) during at least one visit while 28% (9/32) of women demonstrate colonization resistance, even during product administration. Immediately prior to LACTIN-V administration, the colonization-resistant group exhibited elevated vaginal microbiota diversity. During LACTIN-V administration, colonization resistance was associated with elevated vaginal markers of epithelial disruption and reduced chemokines, possibly due to elevated absolute abundance of BV-associated species and reduced *L. crispatus*. Colonization permissive women were stratified into sustained and transient colonization groups (31% and 41% of participants, respectively) based on CTV-05 colonization after cessation of product administration. These groups also exhibited distinct genital immune profiles during LACTIN-V administration.

**Conclusions:**

The genital immune impact of LACTIN-V may be contingent on the CTV-05 colonization phenotype, which is in turn partially dependent on the success of BV clearance prior to LACTIN-V administration.

**Supplementary Information:**

The online version contains supplementary material available at 10.1186/s40168-024-01828-7.

## Introduction

Bacterial vaginosis (BV) is linked to increased risk of multiple adverse reproductive health outcomes, including an increased risk of HIV acquisition [1]. BV is characterized by an increased diversity of vaginal bacteria, including species such as *Gardnerella vaginalis*, and by a lack of protective *Lactobacillus* species, such as *L. crispatus* [2]. The standard of care for BV treatment is a 1-week course of oral or topical antibiotics (metronidazole or clindamycin), but BV recurrence rates are very high, nearing 60% by 12 months post-treatment [3]. *Lactobacillus*-based probiotics and live biotherapeutics, either alone or in combination with antibiotics, have been proposed as alternative treatment strategies. While the success of these approaches has varied, topical application of LACTIN-V, an *L. crispatus*-based live biotherapeutic, was recently shown to reduce BV recurrence rates after metronidazole treatment by 34% compared to matched placebo in a phase 2b, randomized, placebo-controlled trial [4].

BV increases HIV risk in part by eliciting genital mucosal inflammation, which facilitates HIV infection by disrupting the genital epithelial barrier and by recruiting highly HIV-susceptible target cells (e.g., activated CD4 + T cells) to the genital mucosa [1, 5]. Standard BV treatment rapidly reduces genital pro-inflammatory cytokines due to reductions in BV-associated bacteria, and increases the genital level of some chemokines, perhaps due to an increased abundance of *Lactobacillus* species [6, 7]. LACTIN-V administration following standard antibiotic treatment provided sustained reductions in genital inflammation and epithelial disruption 3 months after the last dose of LACTIN-V [8], benefits that were apparent even though 48% of participants in the LACTIN-V arm exhibited sustained colonization by *L. crispatus* CTV-05, the strain present in LACTIN-V, at 3 months after the last dose of LACTIN-V. Here, we attempt to define predictors of colonization by CTV-05 and examine whether the genital immune impact of LACTIN-V differs based on colonization success.

## Methods

### Study participants

**Fig. 1:**
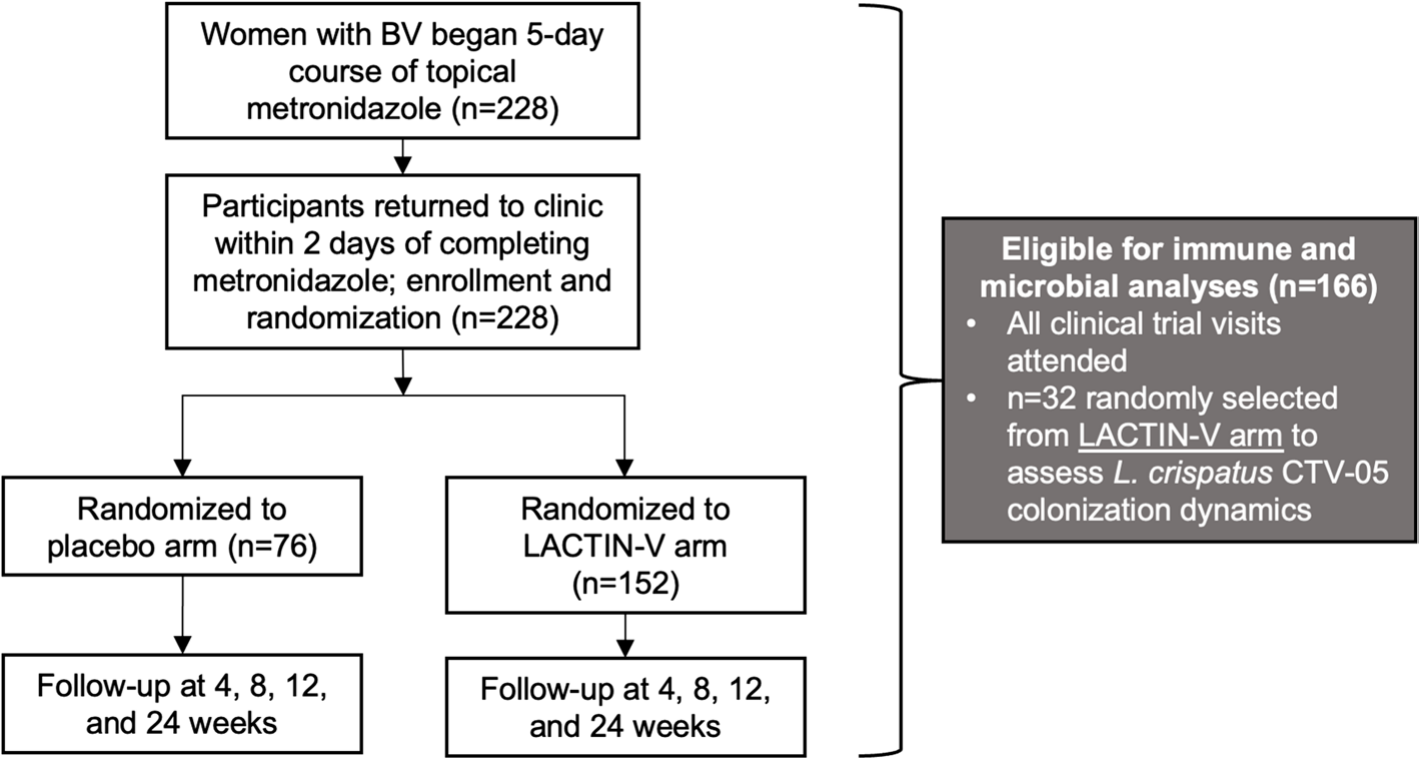
Summary of study design

Participants were recruited into a previously described phase 2b randomized, placebo-controlled clinical trial of the *L. crispatus*-based live biotherapeutic (LACTIN-V) to prevent BV recurrence following standard antibiotic treatment [4]. Two hundred and twenty-eight women were enrolled in the larger clinical trial. Of the 151 participants who received LACTIN-V in the larger clinical trial, we randomly selected a subset of 32 participants who attended all clinical trial visits for the current sub-study (Fig. 1). Sociodemographic factors did not significantly differ between participants included in this sub-analysis and all other participants randomized to receive LACTIN-V (Table S1). Clinical trial details have been outlined in previous publications [4, 7, 8]. Briefly, women with BV (as defined by ≥ 3 Amsel criteria and a Nugent score ≥ 4) at the screening visit, henceforth referred to as the baseline visit, were given a 5-day course of topical metronidazole and then randomized 2:1 to either LACTIN-V or placebo which were administered intravaginally once daily for 5 days followed by twice weekly for 10 weeks. Vaginal swabs were collected at baseline and within 48 h of completing metronidazole treatment (i.e., at enrollment) and at 4 weeks, 8 weeks, 12 weeks (i.e., 1 week after the last dose of LACTIN-V), and 24 weeks (i.e., 13 weeks after the last dose of LACTIN-V) after enrollment. For simplicity, we will refer to the 4-week, 8-week, and 12-week visits as “during” LACTIN-V administration. Immediately following collection, vaginal swabs were plunged into 2 mL of Starplex transport medium, immediately frozen at − 20 °C, and then transferred to a − 80 °C freezer at the end of the day. Behavioral characteristics were also recorded at each study visit.

### Quantification of soluble immune factors

Cervicovaginal swabs were thawed and the remaining eluent (i.e., the transport medium that the swab was plunged into) was centrifuged at 4500 rpm for 30 min. Supernatant was then removed for immune factor analysis and the bacterial pellet was left intact for qPCR analyses. The soluble immune factors IL-1α, IFN-α2A, IL-17A, IL-6, IP-10, IL-8, MIP-1β, MIP-3α, MIG, sE-cad, and MMP-9 were measured in duplicate on the MSD platform according to manufacturer’s instructions as previously described [9] (Meso Scale Discovery, Rockville, MD).

### DNA extraction and quantitative polymerase chain reaction

DNA was extracted from 175µL of the bacterial pellet in vaginal swab samples using the Qiagen DNEasy PowerSoil kit (Qiagen) according to manufacturer’s instructions. Targeted quantitative polymerase chain reaction was used to estimate total bacterial abundance by targeting the 16S region of the rRNA gene and the absolute abundances of *Lactobacillus* species (*L. crispatus*, *L. iners*, *L. jensenii*, and *L. gasseri*) and four common BV-associated bacterial taxa (*G. vaginalis*, *Atopobium vaginae*, *Megasphaera* species, and *Prevotella* species) using previously validated primer and probe sequences described below. All quantitative polymerase chain reaction assays were Taqman-based and performed on the QuantStudio 6 Flex Real-Time PCR System (Thermofisher). Total bacterial load was quantified with qPCR adopted from Nadkarni and colleagues [10]. Protocol for quantification of *L. crispatus*, *L. iners*, *L. jensenii*, and *L. gasseri* absolute abundances with multiplex quantitative polymerase chain reaction was adopted from Balashov and colleagues [11]. Absolute abundances of *G. vaginalis*, *A. vaginae*, and *Megasphaera* spp. were quantified with multiplex quantitative polymerase chain reaction according to Kusters and colleagues [12]. Total *Prevotella* absolute abundance was quantified with quantitative polymerase chain reaction adopted from Martin and colleagues [13]. Primer and probe sequences are presented in Table S2. Total reaction volume for assays was 10µL. Assays for total *Prevotella*, *L. crispatus*, *L. iners*, *L. jensenii*, and *L. gasseri* were performed at 95 °C for 10 min, 45 cycles at 95 °C for 15 s, then 60 °C for 1 min. Assays for *G. vaginalis*, *A. vaginae*, and *Megasphaera* spp. were performed at 95 °C for 10 min, 45 cycles at 95 °C for 15 s, then 55 °C for 1 min. Data analysis was performed with QuantStudio Real-Time PCR Software version 1.3 (Applied Biosystems). Copy numbers were quantified using the following equation, where ΔCt represents the difference in Ct between a sample and negative control: $Copy number={2}^{\Delta Ct}$.

### *L. crispatus* CTV-05 polymerase chain reaction

Polymerase chain reaction (PCR) reactions performed at the University of California San Francisco were set up in duplicate with 25µL QuantiTect SYBR Green RT-PCR Master Mix (Qiagen), 2.5µL forward primer (10uM), 2.5 µL reverse primer (10uM), 10µL extract, and 10µL H2O for a total volume of 50µL. Following initial denaturation at 95 °C for 15 min, PCR cycling consisted of 40 cycles at 95 °C for 30 s, 58 °C for 60 s, and 72 °C for 60 s. Gene targets for strain and species-specific PCR were selected using genes identified in *L. crispatus* CTV-05-specific regions which were absent in other sequenced bacterial strains and with low numbers of homologs in vaginal metagenome datasets. Primer sequences are presented in Table S3. Bacterial concentration was calculated from mean sample Ct values using a standard curve based on serial dilutions of CTV-05 strain of *L. crispatus*. Limits of detection determined at the 95% detection threshold were 6.0 × 10^2^ CFU/mL. Detection *L. crispatus* CTV-05 was defined as above the lower limit of detection as defined above. A concentration of *L. crispatus* CTV-05 greater than 10^6^ CFU/mL was pre-defined as the threshold for clinically relevant colonization, based on published evidence that this concentration of *L. crispatus* CTV-05 was associated with reduced recurrence of BV [4] and UTIs [14, 15].

### DNA extraction and metagenomic sequencing

Samples were plated from swab collection tubes into ZymoBIOMICS lysis solution for DNA extraction. DNA was extracted and processed with high-throughput automation liquid handlers (Agilent Bravo system and Labcyte ECHO instrument) to maintain constancy in sample experimentation and decrease laboratory processing time. The ZymoBIOMICS 96 MagBead DNA kit was followed as instructed to extract DNA from swab samples, water controls, and storage medium controls. Illumina library preparation was performed using a miniaturized protocol of NEBNext Ultra II FS DNA Library Prep kit for DNA for the Labcyte ECHO instrument [16]. More than 25 million paired-end 150 bp reads per patient sample were collected on an Illumina NovaSeq instrument. The CZ ID platform was used to process raw sequencing reads and remove host reads [17]. The VIRGO bioinformatic pipeline was used to align processed reads with established databases to identify microbes and inferred microbial functions [18]. Samples with more than 100,000 microbial reads were included in the present analyses. Inferred microbial functions were quantified by calculating the relative abundance of genes mapping to individual KEGG orthologs [19].

### Statistical analysis

Soluble immune factor levels and gene copy numbers were normalized through log_10_-transformation. Comparison of *L. crispatus* CTV-05 absolute abundance between colonization was performed with the Mann–Whitney *U* test. The Pearson Chi-Square test or Mann–Whitney *U* test was used to compare the frequency (if dichotomous) or value (if continuous) of sociodemographic factors and sexual behaviors between colonization groups. Clustering on pre-LACTIN-V immune data only included participants with complete immune data at the pre-LACTIN-V visit and was performed with k means clustering using the *kmeans* function in the “stats” package in R. Optimal number of clusters for k means clustering was determined using silhouette analysis with the *fviz_nbclust* function in the “factoextra” package in R. Principal component analysis (PCA) plots were generated using the *prcomp* function and visualized with the *fviz_pca_biplot* function in the “factoextra” and “FactoMineR” packages in R. Principal coordinate analysis (PCoA) plots were generated using a Bray–Curtis dissimilarity matrix that was constructed using the relative abundance of all bacteria species identified with metagenomic sequencing prior to LACTIN-V administration. Comparison of overall vaginal microbiota composition prior to LACTIN-V administration was performed with ANOSIM. PCoA and ANOSIM analyses were performed using the “vegan” and “dplyr” R packages and PCoA visualization was performed with the “ggplot2” R package. Shannon diversity of the vaginal microbiota was determined at the species level using the “diversity” function in the “vegan” package in R with default parameters. Differential abundance analysis of the relative abundance of bacterial species prior to LACTIN-V administration was performed with linear discriminant analysis effect size (LEfSe) [20]. Since we did not include a subclass comparison in our LEfSe analysis, we adjusted *p* values with the false discovery rate [21]. Differential abundance analysis of KEGG orthologs was performed with MaAsLin2 and *p* values were adjusted for multiple comparisons with the false discovery rate [22]. Comparisons of levels of immune factors and bacterial absolute abundances and Shannon diversity of the vaginal microbiota prior to LACTIN-V administration between colonization groups were performed with the Mann–Whitney *U* test. Linear mixed models were generated to evaluate the association between immune factors and bacterial abundances at each visit during LACTIN-V administration. Participants IDs were included as random effects in each model to allow for the inclusion of repeated measurements without violating the assumption of independence. The association between immune factors and bacterial absolute abundances during LACTIN-V administration was performed with linear mixed models that included participant IDs as random effects. Comparisons of immune factors and bacterial abundances at 24 weeks (i.e., 13 weeks after the last dose of LACTIN-V) between sustained colonizers, transient colonizers, and colonization-resistant women were performed with ANOVAs and pairwise comparisons were performed with the Tukey post hoc test. All statistical tests were performed with RStudio (version 2022.02.3) or GraphPad Prism (version 9.0.2).

## Results

### Distinct patterns of *L. crispatus* CTV-05 colonization after LACTIN-V administration

**Fig. 2:**
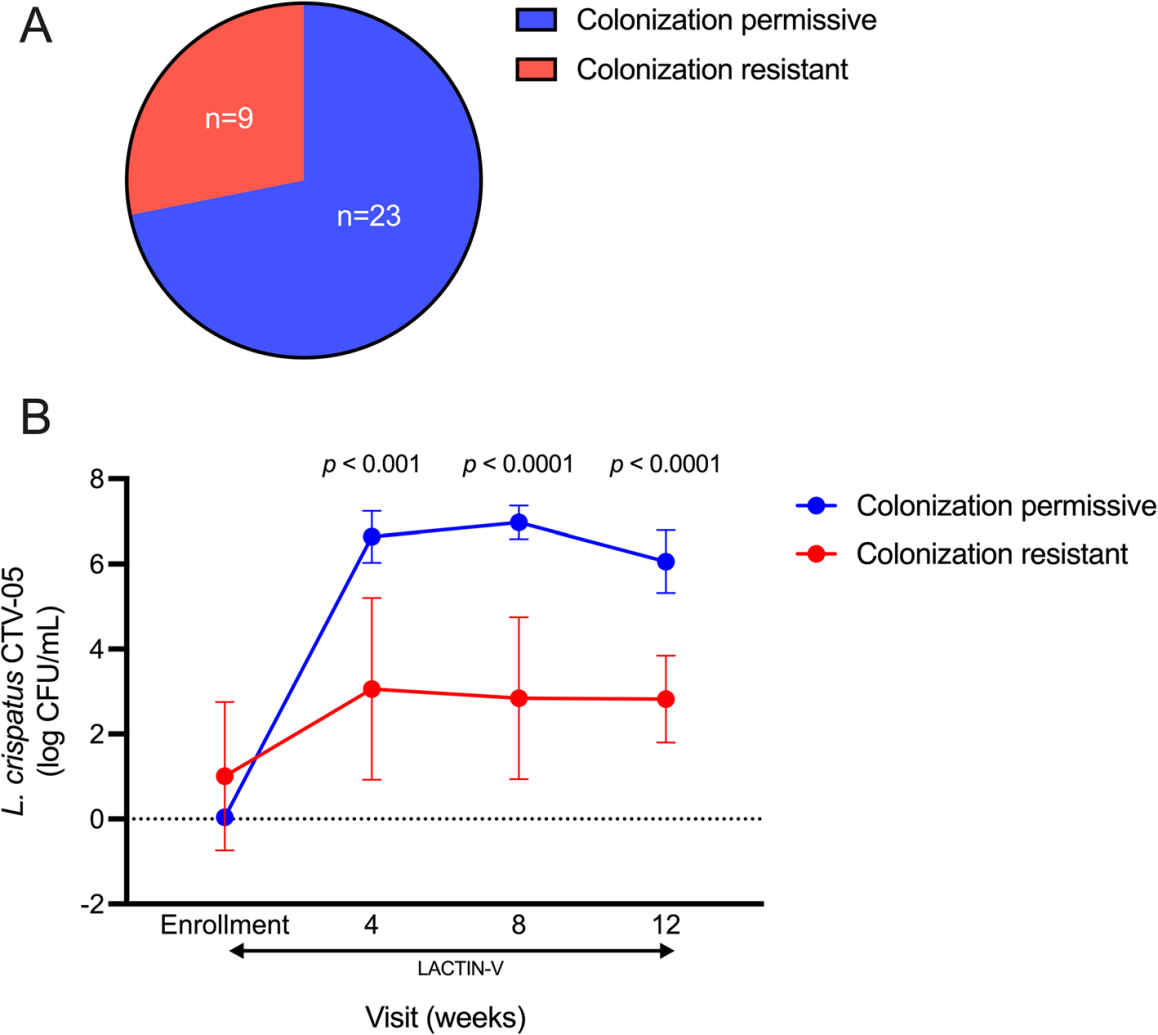
Resistance to *L. crispatus* CTV-05 colonization observed among subset of participants. **A** Proportion of colonization-resistant and permissive participants observed among women who received LACTIN-V. **B** Comparison of the absolute abundance of *L. crispatus* CTV-05 at each visit during LACTIN-V administration between colonization-resistant and permissive women (*n* = 32, Mann–Whitney *U* test). Data points and error bars are mean and 95% confidence intervals, respectively

Clinically relevant *L. crispatus* CTV-05 colonization was present during at least one study visit for 23 participants (72%); colonization resistance, defined as the absence of clinically relevant *L. crispatus* CTV-05 colonization at every visit, was observed in 9 participants (28%). By definition, the colonization-resistant group exhibited significantly lower *L. crispatus* CTV-05 absolute abundance during and after LACTIN-V administration (Figs. 2 and S1). Among women who exhibited CTV-05 colonization permissiveness (*n* = 23), transient colonization was defined as the detection of clinically relevant CTV-05 colonization only during application of the LACTIN-V product (*n* = 13; 41%) while sustained colonization was defined as the detection of clinically relevant CTV-05 colonization for at least 3 months after LACTIN-V cessation (*n* = 10; 31%).

### Resistance to colonization by *L. crispatus* CTV-05 is associated with elevated preceding vaginal microbiota diversity

**Fig. 3:**
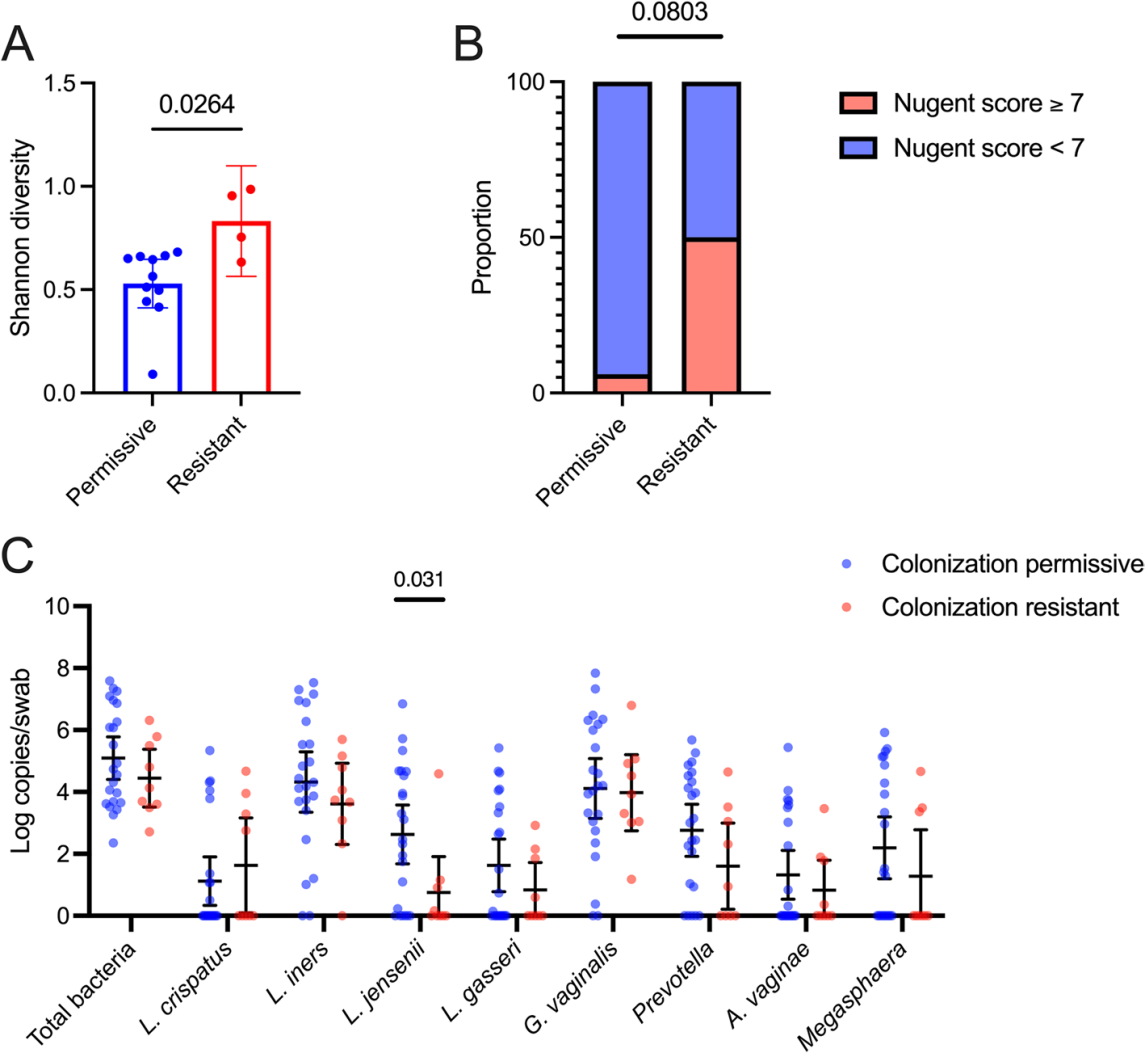
Elevated vaginal microbiota diversity following metronidazole treatment among colonization resistant women. **A** Difference in Shannon diversity of the vaginal microbiota prior to LACTIN-V administration between colonization-resistant and permissive women (*n* = 15, Mann–Whitney *U* test). **B** Rates of Nugent BV within 48 h of completing metronidazole treatment between colonization permissive and resistant groups (*n* = 22, Pearson Chi-Square test). **C** Comparison of bacterial absolute abundances immediately prior to LACTIN-V administration based on colonization permissiveness or resistance (*n* = 32, Mann–Whitney *U* test). Data points and error bars are mean and 95% confidence intervals, respectively

We first sought to identify predictors of *L. crispatus* CTV-05 colonization resistance. Neither the number of missed doses of LACTIN-V, sociodemographic factors, nor self-reported sexual behaviors before or during LACTIN-V administration differed based on colonization phenotype (Table S4). We then focused on the timepoint immediately after metronidazole treatment but prior to administration of the first LACTIN-V dose (within 48 h of completing metronidazole treatment) in order to assess pre-application microbial or immune predictors of subsequent colonization resistance. Sufficient metagenomic reads (> 100,000 microbial reads) were available for 11 participants in the colonization permissive group and 4 participants in the colonization resistant group. We did not identify any differentially abundant bacterial species based on linear discriminant analysis effect size (LEfSe) after adjusting for multiple comparisons with false discovery rate (Table S5). However, among the colonization-resistant group, the pre-application vaginal microbiota Shannon diversity was significantly higher (*p* = 0.0264), rates of Nugent BV (defined as Nugent score ≥ 7) tended to be higher (*p* = 0.0803), and the absolute abundance of *L. jensenii* (Fig. 3; *p* = 0.0306) was significantly lower. We also compared inferred microbial function (represented by KEGG orthologs) between colonization permissive and resistant groups but did not identify any differentially abundant functions after correcting for multiple comparisons (Table S6). There was no association of vaginal soluble immune factor levels pre-LACTIN-V administration with subsequent colonization phenotype (Figure S2). To test whether the overall genital immune milieu, rather than individual immune factors, prior to LACTIN-V administration predicted CTV-05 colonization status, we generated pre-LACTIN-V immune clusters with k means clustering for all women with complete immune data at this visit (*n* = 31). Silhouette analysis determined that the optimal number of clusters was two, although the proportion of women belonging to each immune cluster did not differ between colonization permissive and resistant women (Fig. S2). Given the substantial heterogeneity in CTV-05 absolute abundance in the colonization-resistant group, especially at the 4-week visit (i.e., the first visit after the start of LACTIN-V administration), we also explored pre-LACTIN-V predictors of CTV-05 absolute abundance at the 4-week visit in the colonization-resistant group. There was no association between pre-LACTIN-V Shannon diversity or bacterial absolute abundances and CTV-05 absolute abundance in the colonization resistant group (Fig. S3).

### *L. crispatus* CTV-05 colonization resistance modulates the genital immune and microbial impact of LACTIN-V application

**Fig. 4:**
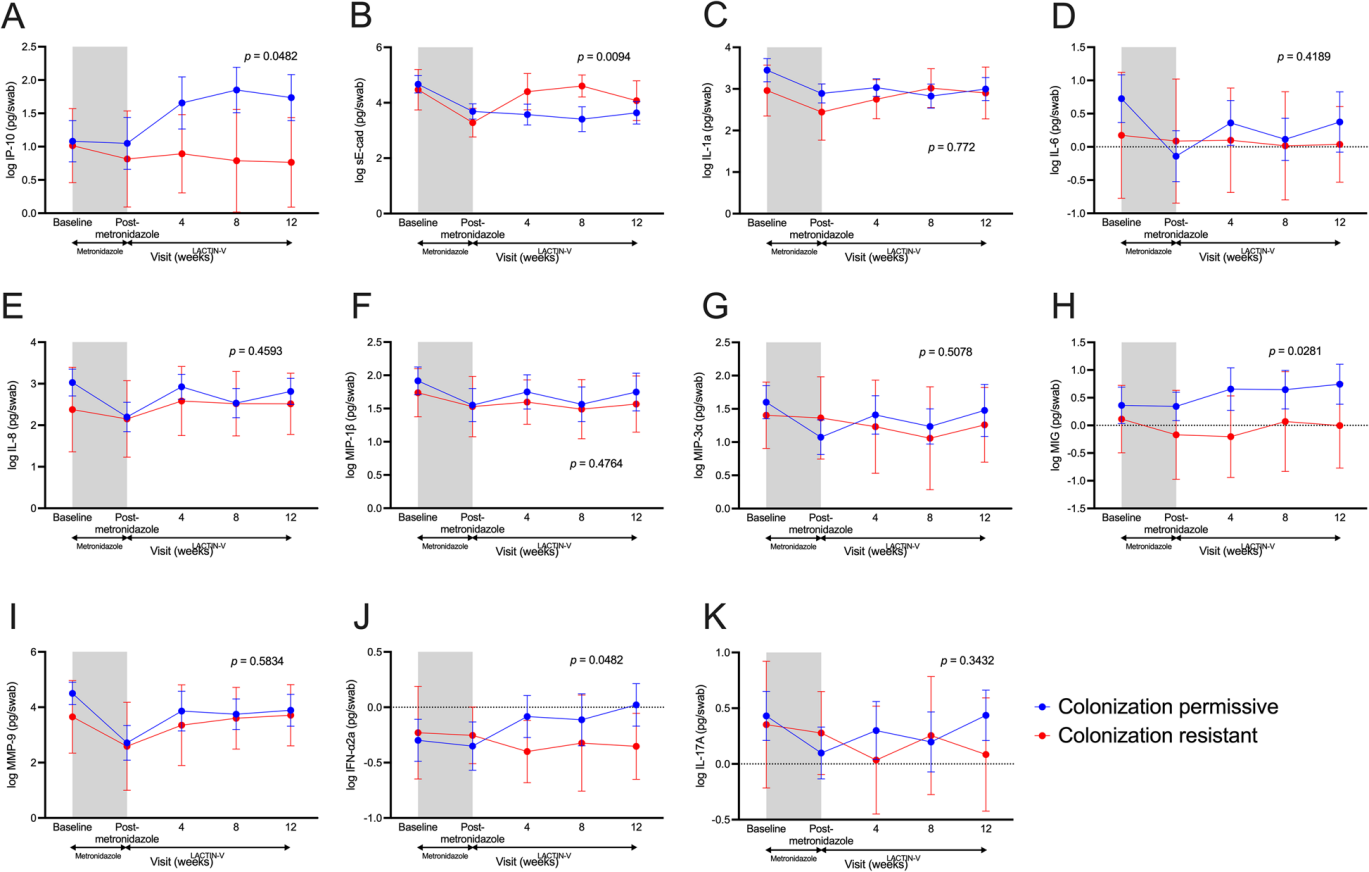
Distinct genital immune profiles during LACTIN-V administration between colonization-resistant and permissive women. Vaginal levels of **A** IP-10, **B** sE-cad, **C** IL-1a, **D** IL-6, **E** IL-8, **F** MIP-1b, **G** MIP-3a, **H** MIG, **I** MMP-9, **J** IFN-a2a, and **K** IL-17A over the course of the study between colonization-resistant and permissive women. Shaded area represents time during metronidazole treatment which was not included in analyses. *p* values determined with linear mixed models including all time points during LACTIN-V administration (*n* = 32). Data points and error bars are mean and 95% confidence intervals, respectively

**Fig. 5:**
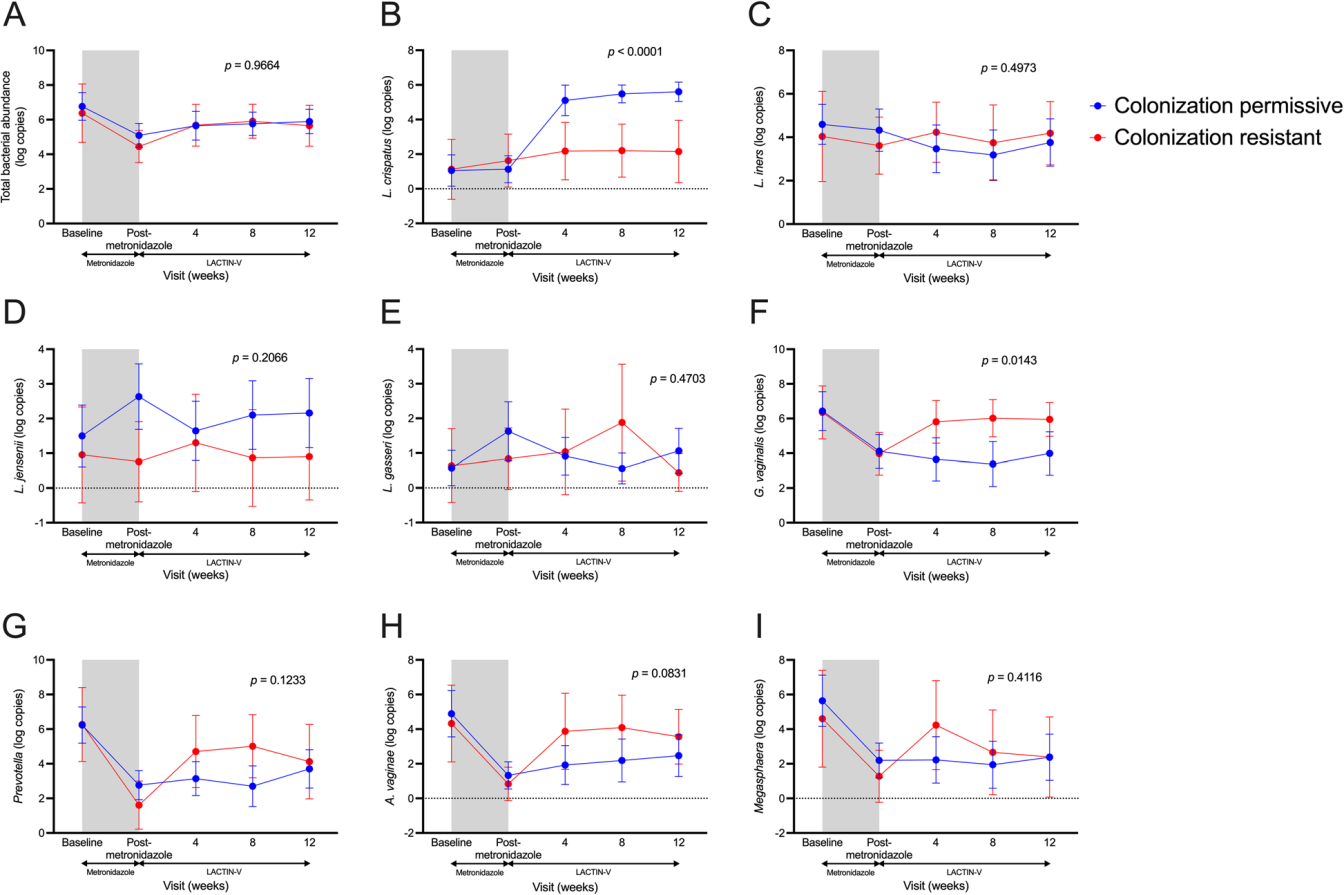
Absolute abundance of key vaginal bacterial taxa differ based on *L. crispatus* CTV-05 colonization resistance. Vaginal **A** total bacterial load and absolute abundance of **B** *L. crispatus*, **C** *L. iners*, **D** *L. jensenii*, **E** *L. gasseri*, **F** *G. vaginalis*, **G** *Prevotella* spp., **H** *A. vaginae*, and **I** *Megasphaera* spp. between colonization-resistant and permissive women over the course of the study. Shaded area represents time during metronidazole treatment which was not included in analyses. *p* values determined with linear mixed models including data from all visits during LACTIN-V administration (*n* = 32). Data points and error bars are mean and 95% confidence intervals, respectively

We next assessed the impact of CTV-05 colonization phenotype on genital immunology and/or microbiota during LACTIN-V administration. We generated linear mixed models to assess potential differences in genital immune factors and the absolute abundance of vaginal bacterial taxa during LACTIN-V administration between colonization-resistant and permissive women. During LACTIN-V administration, colonization-resistant women exhibited significantly elevated sE-cad (*p* = 0.0094) and reduced IP-10 (*p* = 0.0482), MIG (*p* = 0.0281), and IFN-α2a (Fig. 4; *p* = 0.0482) compared to colonization permissive women. We also observed a reduced *L. crispatus* absolute abundance (*p* < 0.0001), as expected, and an elevated absolute abundance of *G. vaginalis* (*p* = 0.0143) among colonization-resistant women (Fig. 5).

Given the strong links between the vaginal microbiota and genital immunology, we hypothesized that the genital immune differences observed between colonization-resistant and permissive women would be driven by the differences in the absolute abundance of vaginal bacterial taxa. In linear mixed models that incorporated measurements from samples obtained during LACTIN-V administration, *L. crispatus* absolute abundance was associated with elevated IP-10 (*p* < 0.0001), MIG (*p* < 0.0001), and IFN-α2a (*p* = 0.0057), and *G. vaginalis* absolute abundance was associated with elevated sE-cad (*p* < 0. 0001; Table 1 and Fig. S4). To explore potential microbial functions underpinning these associations, we also compared KEGG orthologs between colonization permissive and resistant groups during LACTIN-V administration but did not identify any differentially abundant functions (Table S7–9). In summary, CTV-05 colonization resistance was associated with a distinct genital immune profile compared to colonization permissiveness during LACTIN-V administration, which was driven by elevated *L. crispatus* absolute abundance and a reduced absolute abundance of BV-associated bacteria.

**Table 1:** Associations between genital immune factors and bacterial absolute abundances linked with *L. crispatus* CTV-05 colonization resistance/permissiveness during LACTIN-V administration

	sE-cad	IP-10	MIG	IFN-α2a
*L. crispatus*	− 0.07 (0.1346)	**0.23 (< 0.0001)**	**0.21 (< 0.0001)**	**0.07 (0.0057)**
*G. vaginalis*	**0.21 (< 0.0001)**	− 0.05 (0.145)	− 0.02 (0.4762)	− 0.01 (0.7182)

Data are linear mixed model estimate (*p* value)

*p* values generated from Tukey post hoc tests performed on linear mixed model analyses (*n* = 32)

### Sustained vs. transient *L. crispatus* CTV-05 colonization phenotypes are associated with distinct genital immune profiles during LACTIN-V administration

**Fig. 6:**
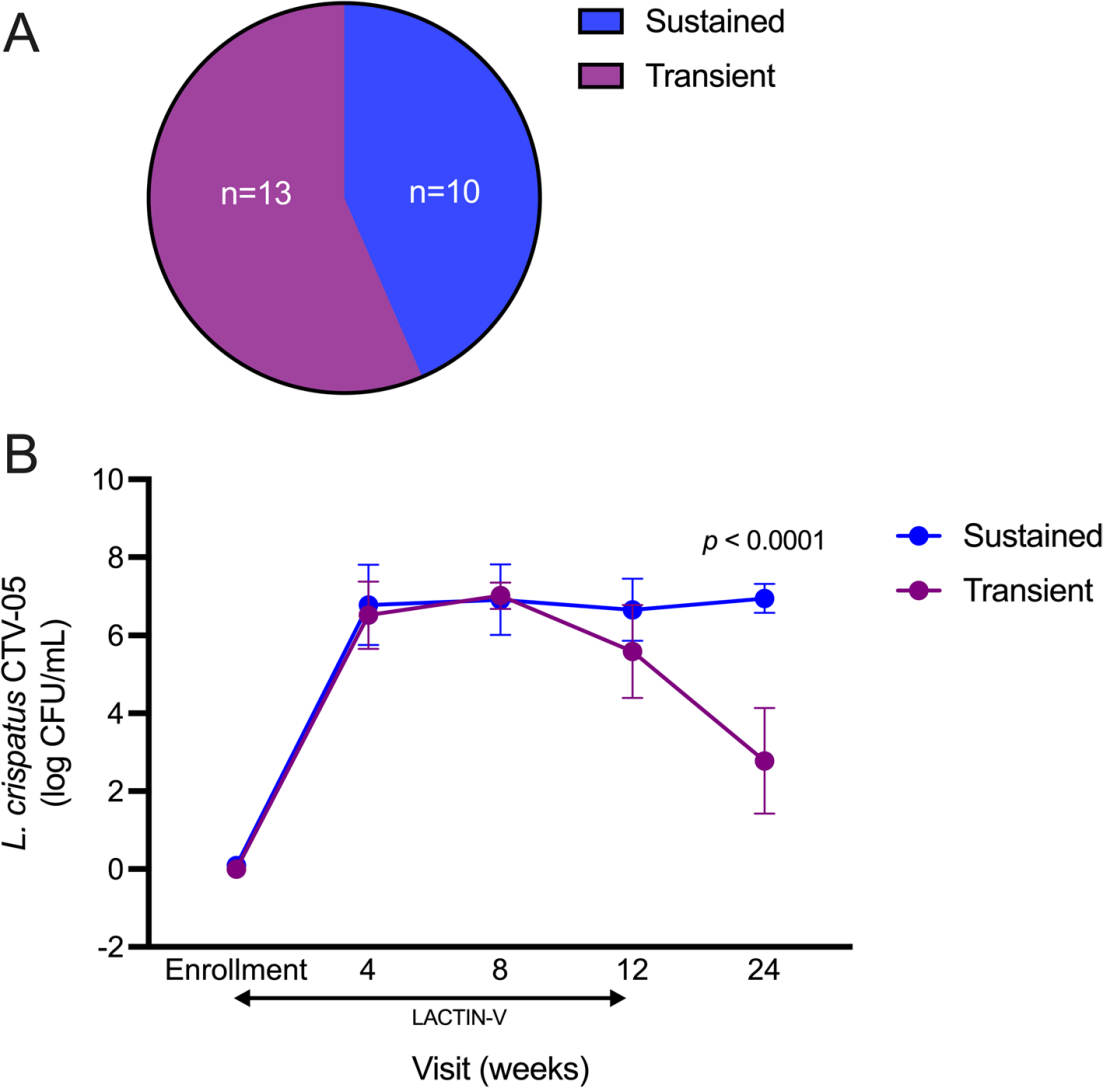
Sustained and transient *L. crispatus* CTV-05 colonization observed among colonization permissive women. **A** Proportion of colonization permissive women who exhibited sustained or transient *L. crispatus* CTV-05 colonization. **B** Comparison of the absolute abundance of *L. crispatus* CTV-05 at each visit between sustained and transient colonizers (*n* = 23, Mann–Whitney *U* test). Data points and error bars are mean and 95% confidence intervals, respectively

**Fig. 7:**
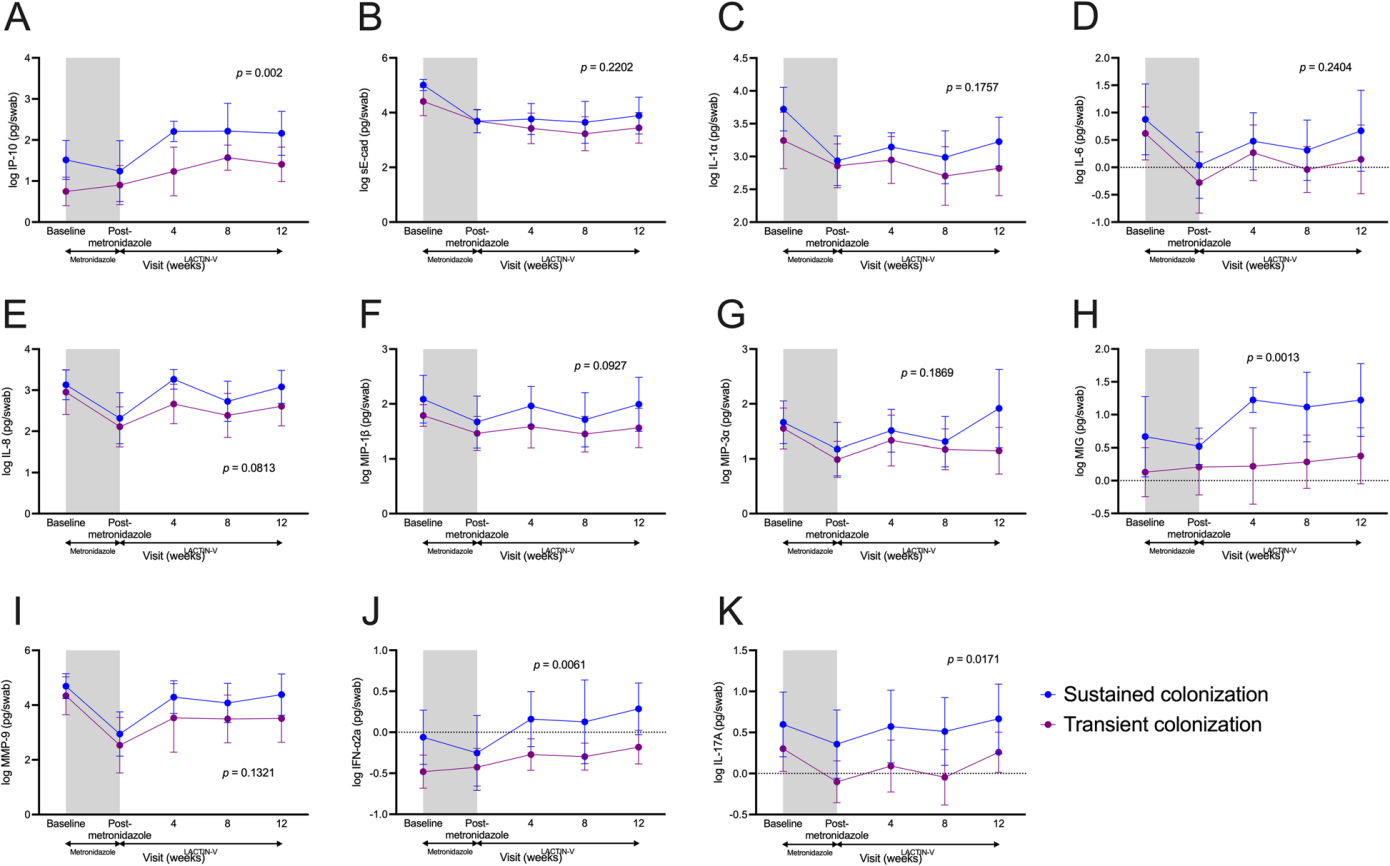
Sustained and transient colonization groups exhibit distinct genital immune profiles during LACTIN-V administration. Vaginal levels of **A** IP-10, **B** sE-cad, **C** IL-1α, **D** IL-6, **E** IL-8, **F** MIP-1β, **G** MIP-3α, **H** MIG, **I** MMP-9, **J** IFN-α2a, and **K** IL-17A over the course of the study based on sustained and transient colonization. Shaded area represents time during metronidazole treatment which was not included in analyses. *p* values determined with linear mixed models including all time points during LACTIN-V administration (*n* = 23). Data points and error bars are mean and 95% confidence intervals, respectively

**Fig. 8:**
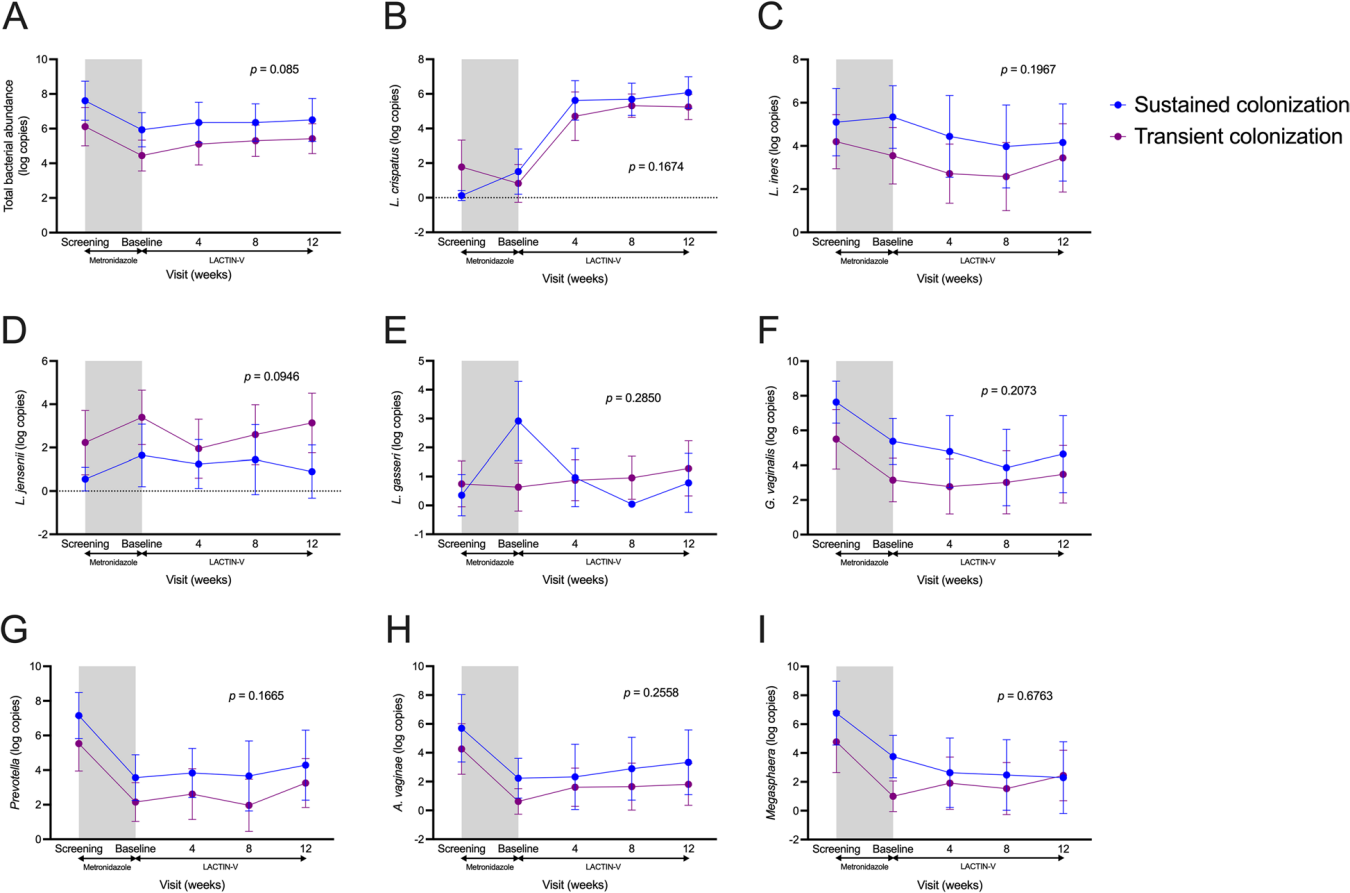
No difference in key bacteria species during LACTIN-V administration between sustained and transient colonization groups. Vaginal **A** total bacterial load and absolute abundance of **B** *L. crispatus*, **C** *L. iners*, **D** *L. jensenii*, **E** *L. gasseri*, **F** *G. vaginalis*, **G** *Prevotella* spp., **H** *A. vaginae*, and **I** *Megasphaera* spp. based on sustained and transient colonization over the course of the study. Shaded area represents time during metronidazole treatment which was not included in analyses. *p* values determined with linear mixed models including data from all visits during LACTIN-V administration (*n* = 23). Data points and error bars are mean and 95% confidence intervals, respectively

During LACTIN-V administration, the absolute vaginal abundance of *L. crispatus* CTV-05 was similar for participants who went on to display sustained vs. transient colonization after product cessation, but (by definition) when assayed 3 months after product discontinuation *L. crispatus* CTV-05 abundance was significantly higher among women with sustained colonization (Fig. 6). Sustained vs. transient colonization phenotypes were not associated with differences in the number of missed doses of LACTIN-V, in sociodemographic factors, or in measured sexual behaviors (Table S10). Although the absolute abundance of *L. crispatus* CTV-05 was similar in sustained and transient colonization groups during LACTIN-V administration, we were interested to assess possible differences in genital immunology or the absolute abundance of key endogenous vaginal bacteria during product administration. Linear mixed models were generated to evaluate the association between sustained/transient colonization and levels of genital immune factors and the absolute abundance of key vaginal bacterial taxa during LACTIN-V administration. The sustained colonization group exhibited significantly greater IP-10 (*p* = 0.002), MIG (*p* = 0.0013), IFN-a2a (*p* = 0.0061), and IL-17A (*p* = 0.0171; Fig. 7) during LACTIN-V administration compared to the transient colonization group, but no differences were observed between groups in the absolute abundance of other bacterial taxa (Fig. 8).

### Elevated total bacterial abundance prior to LACTIN-V administration in sustained colonization group

**Fig. 9:**
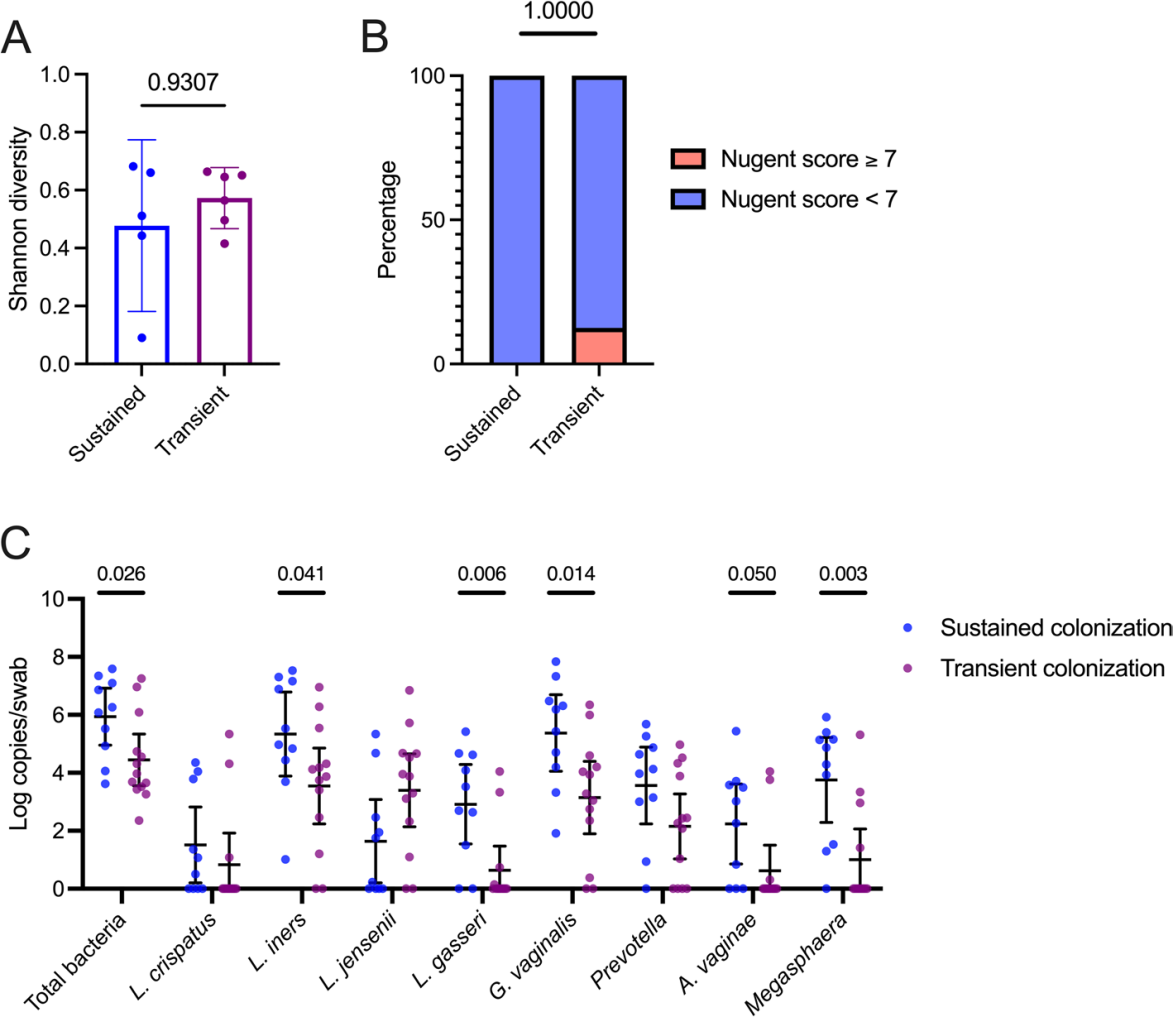
Elevated vaginal bacterial absolute abundance, but not diversity, among sustained colonization group prior to LACTIN-V administration. **A** Difference in Shannon diversity of the vaginal microbiota prior to LACTIN-V administration based on sustained and transient colonization (*n* = 11, Mann–Whitney *U* test). **B** Rates of Nugent BV within 48 h of completing metronidazole treatment between sustained and transient colonization groups (*n* = 16, Fisher’s exact test). **C** Difference in bacterial absolute abundances immediately prior to LACTIN-V administration between sustained and transient colonization groups (*n* = 23, Mann–Whitney *U* test). Data points and error bars are mean and 95% confidence intervals, respectively

Since the sustained and transient colonization groups exhibited different genital immune profiles during LACTIN-V administration, we next focused on the timepoint immediately after metronidazole treatment but prior to LACTIN-V administration, in order to assess potential predictors of sustained vs. transient colonization. Prior to LACTIN-V administration, there was no difference in vaginal microbiota Shannon diversity between groups (Fig. 9) or differences in bacterial relative abundances after controlling for multiple comparisons (Table S11). There were also no immune differences between the sustained and transient colonization groups prior to LACTIN-V administration (Fig. S5). However, the group that went on to demonstrate sustained *L. crispatus* CTV-05 colonization had elevated bacterial abundance in a non-specific manner, including greater total bacterial load (*p* = 0.02552) and a greater absolute abundance of several different bacteria including *L. iners* (*p* = 0.04379), *L. gasseri* (*p* = 0.007804), *G. vaginalis* (*p* = 0.01693)*, A. vaginae* (*p* = 0.05098), and *Megasphaera* (*p* = 0.004409; Fig. 9). These microbial differences also extended to the pre-metronidazole timepoint, when the sustained colonization group exhibited greater total bacterial density (*p* = 0.04216) and *G. vaginalis* absolute abundance (*p* = 0.04379), as well as higher IFN-a2a (*p* = 0.03154), IP-10 (*p* = 0.01778), and a tendency towards greater sE-cad (*p* = 0.05746; Fig. S6).

### Impact of colonization phenotype on vaginal immunology and microbiota after LACTIN-V product cessation

LACTIN-V administration after BV treatment has been shown to provide sustained immune benefits compared to placebo [8], and so we next explored whether these sustained differences in vaginal immunology were driven by the three *L. crispatus* CTV-05 colonization phenotypes (sustained colonization, transient colonization, and colonization resistance). Colonization phenotype was not associated with significant differences in genital immune factors 3 months after product cessation (Fig. S7). The sustained colonization group exhibited a greater absolute abundance of *L. crispatus* compared to the transient and resistant colonization groups (*p* = 0.0288 and *p* = 0.0002, respectively) and also had a reduced *G. vaginalis* absolute abundance compared to the resistant group (Fig. S8; *p* = 0.0353).

## Discussion

Vaginal microbiota predominance by *L. crispatus*, as opposed to a BV-type microbiota, is associated with protection against adverse reproductive health outcomes, including STI and HIV acquisition. This protection is thought to be mediated by reduced levels of genital inflammation [5]. Vaginal application of LACTIN-V following topical metronidazole treatment of BV reduced subsequent recurrence, and was associated with reduced genital inflammation and epithelial disruption 3 months after the last dose of LACTIN-V [4, 8]. However, sustained detection of *L. crispatus* CTV-05 was seen in 48% of trial participants [4]. In the current study, we explored whether patterns of clinically relevant vaginal CTV-05 colonization altered the genital immune impact of LACTIN-V in a subset of trial participants. Resistance to CTV-05 colonization, even during product administration, was seen in almost a third of participants and was associated with higher pre-application vaginal microbiota diversity. Furthermore, during LACTIN-V administration, there were clear genital immune differences based on colonization permissiveness, which were driven by differences in the abundance of key vaginal bacteria. In addition, sustained CTV-05 colonization after discontinuation of LACTIN-V administration was only observed in a subset of these colonization permissive women and was also associated with immune differences compared to the transient colonization phenotype.

In these analyses, we defined at least 10^6^ CFU/mL of *L. crispatus* CTV-05 as the a priori cut off for clinically relevant colonization, based on previously published evidence that this concentration of CTV-05 is associated with protection against recurrent urinary tract infections and BV [15]. Using this definition, we demonstrated that a subset of women was resistant to *L. crispatus* CTV-05 colonization even during LACTIN-V administration, a phenotype that we defined as colonization resistance. In contrast with previous studies, we did not observe any association between colonization resistance and sexual behaviors [23, 24], although there was elevated vaginal microbiota diversity immediately after metronidazole treatment (and just prior to LACTIN-V administration) among the colonization-resistant group. Colonization-resistant and permissive women also exhibited distinct genital immune profiles and different absolute abundances of key vaginal bacteria during LACTIN-V administration, suggesting that the genital immune impact of LACTIN-V may be contingent on CTV-05 colonization, which is hindered by poor BV clearance prior to LACTIN-V administration.

Among the colonization permissive group, we observed two distinct phenotypes, defined by subsequent sustained CTV-05 colonization 3 months after the last dose of LACTIN-V (sustained colonization) and CTV-05 colonization only during LACTIN-V administration (transient colonization). Despite having comparable absolute abundances of CTV-05 and other bacteria during LACTIN-V administration, these groups exhibited distinct genital immune profiles. The distinct immune profiles may be a consequence of heightened epithelial adherence and functional output of CTV-05 in the sustained group and passive “flow through” of CTV-05 in the transient group that is not immediately apparent when looking only CTV-05 abundance. However, future studies will be needed to confirm whether CTV-05 activity (e.g., gene transcription and metabolite production) differs based on sustained and transient colonization. In the sustained colonization group, we also observed elevated bacterial abundance immediately prior to LACTIN-V administration and prior to metronidazole treatment in a species-independent manner, including elevated total bacterial density and greater *Lactobacillus* species and BV-associated species. This differs from previous studies which found that CTV-05 colonization was inhibited by endogenous *L. crispatus* [23, 24] and is at odds with evidence that a sparser microbial niche (i.e., a microenvironment with fewer bacteria) is more receptive to colonization by new species [25]. Our finding might be explained by differences in environmental factors that non-specifically enhance bacterial growth among the sustained colonization group, but future metabolomic and proteomic studies are needed to explore this relationship in greater detail.

Three months after the discontinuation of LACTIN-V application, we observed no differences in genital immunology between the three colonization phenotypes, although the sustained colonization group had significantly elevated *L. crispatus* absolute abundance and reduced absolute abundance of *G. vaginalis*. Our relatively low sample size compared to prior analyses may explain the lack of robust immune and microbial differences between colonization phenotypes at 24 weeks and emphasizes the need to explore these effects in larger cohorts.

Adherence to metronidazole treatment was self-reported by participants and was not confirmed by additional laboratory testing. However, the high rates of BV clearance following metronidazole treatment in this cohort, as shown in previous work [7], is consistent with initial BV clearance rates in the literature [26]. Hormone levels were not measured which limited our ability to control for phase of the menstrual cycle, but no study visits occurred during menstruation and all participants were enrolled shortly after menses to roughly control for phase of the menstrual cycle.

Better clinical approaches to optimize the vaginal microbiota are needed to improve reproductive health outcomes. Here, we show that a reduced vaginal microbiota diversity prior to LACTIN-V application was a key determinant of *L. crispatus* CTV-05 colonization, and that the genital immune effects of LACTIN-V were limited to those permissive to CTV-05 colonization, particularly when colonization was sustained after LACTIN-V cessation. Our results suggest that the genital immune effects of LACTIN-V are dependent on CTV-05 colonization, and that clearing a diverse vaginal microbiota prior to LACTIN-V administration may be necessary to ensure CTV-05 colonization.

### Supplementary Information

Supplementary Material 1.

Supplementary Material 2.

Supplementary Material 3.

## Data Availability

All metagenomic sequencing data is available on the NCBI Sequence Read Archive under accession numbers PRJNA784288 and PRJNA1090602.

## References

[1] Anahtar MN, Byrne EH, Doherty KE, Bowman BA, Yamamoto HS, Soumillon M (2015). Cervicovaginal bacteria are a major modulator of host inflammatory responses in the female genital tract. Immunity.

[2] Ravel J, Gajer P, Abdo Z, Schneider GM, Koenig SSK, McCulle SL (2011). Vaginal microbiome of reproductive-age women. PNAS.

[3] Bradshaw CS, Morton AN, Hocking J, Garland SM, Morris MB, Moss LM (2006). High recurrence rates of bacterial vaginosis over the course of 12 months after oral metronidazole therapy and factors associated with recurrence. J Infect Dis.

[4] Cohen CR, Wierzbicki MR, French AL, Morris S, Newmann S, Reno H (2020). Randomized trial of lactin-V to prevent recurrence of bacterial vaginosis. NEJM.

[5] Gosmann C, Anahtar MN, Handley SA, Farcasanu M, Abu-Ali G, Bowman BA (2017). Lactobacillus-deficient cervicovaginal bacterial communities are associated with increased HIV acquisition in young South African women. Immunity.

[6] Joag V, Obila O, Gajer P, Scott MC, Dizzell S, Humphrys M (2018). Impact of standard bacterial vaginosis treatment on the genital microbiota, immune milieu, and ex vivo human immunodeficiency virus susceptibility. Clin Infect Dis.

[7] Armstrong E, Hemmerling A, Miller S, Burke KE, Newmann SJ, Morris SR, et al. Metronidazole treatment rapidly reduces genital inflammation through effects on bacterial vaginosis- associated bacteria rather than lactobacilli. J Clin Invest. 2022;132(6):e152930. 10.1172/JCI152930 PMC8920324 35113809

[8] Armstrong E, Hemmerling A, Miller S, Burke KE, Newmann SJ, Morris SR (2022). Sustained effect of LACTIN-V (Lactobacillus crispatus CTV-05) on genital immunology following standard bacterial vaginosis treatment: results from a randomised, placebo-controlled trial. Lancet Microbe.

[9] Mohammadi A, Bagherichimeh S, Perry MC, Fazel A, Tevlin E, Huibner S, et al. The impact of cervical cytobrush sampling on cervico-vaginal immune parameters and microbiota relevant to HIV susceptibility. Sci Rep. 2020;10:8514. 10.1038/s41598-020-65544-6 PMC7244754 32444843

[10] Nadkarni MA, Martin FE, Jacques NA, Hunter N. Determination of bacterial load by real-time PCR using a broad-range (universal) probe and primers set. Microbiology. 2002;148(1):257–66. 10.1099/00221287-148-1-257 11782518

[11] Balashov SV, Mordechai E, Adelson ME, Sobel JD, Gygax SE (2014). Multiplex quantitative polymerase chain reaction assay for the identification and quantitation of major vaginal lactobacilli. Diagn Microbiol Infect Dis.

[12] Kusters JG, Reuland EA, Bouter S, Koenig P, Dorigo-Zetsma JW (2015). A multiplex real-time PCR assay for routine diagnosis of bacterial vaginosis. Eur J Clin Microbiol Infect Dis.

[13] Martin FE, Nadkarni MA, Jacques NA, Hunter N (2002). Quantitative microbiological study of human carious dentine by culture and real-time PCR: association of anaerobes with histopathological changes in chronic pulpitis. J Clin Microbiol.

[14] Stapleton AE, Au-Yeung M, Hooton TM, Fredricks DN, Roberts PL, Czaja CA, et al. Randomized, placebo-controlled phase 2 trial of a lactobacillus crispatus probiotic given intravaginally for prevention of recurrent urinary tract infection. Clin Infect Dis. 2011;52(10):1212–7. 10.1093/cid/cir183 PMC3079401 21498386

[15] Lagenaur LA, Hemmerling A, Chiu C, Miller S, Lee PP, Cohen CR (2020). Connecting the dots: translating the vaginal microbiome into a drug. J Infect Dis.

[16] Mayday MY, Khan LM, Chow ED, Zinter MS, DeRisi JL. Miniaturization and optimization of 384-well compatible RNA sequencing library preparation. PLoS One. 2019;14(1):e0206194. 10.1371/journal.pone.0206194 PMC6328170 30629604

[17] Kalantar KL, Carvalho T, De Bourcy CFA, Dimitrov B, Dingle G, Egger R, et al. IDseq-an open source cloud-based pipeline and analysis service for metagenomic pathogen detection and monitoring. Gigascience. 2021;9(10):giaa111. 10.1093/gigascience/giaa111 PMC7566497 33057676

[18] Ma B, France MT, Crabtree J, Holm JB, Humphrys MS, Brotman RM, et al. A comprehensive non-redundant gene catalog reveals extensive within-community intraspecies diversity in the human vagina. Nat Commun. 2020;11:940. 10.1038/s41467-020-14677-3 PMC7044274 32103005

[19] Kanehisa M, Goto S, Furumichi M, Tanabe M, Hirakawa M. KEGG for representation and analysis of molecular networks involving diseases and drugs. Nucleic Acids Res. 2009;38:D355–60. 10.1093/nar/gkp896 PMC2808910 19880382

[20] Segata N, Izard J, Waldron L, Gevers D, Miropolsky L, Garrett WS, et al. Metagenomic biomarker discovery and explanation. Genome Biol. 2011;12:R60. 10.1186/gb-2011-12-6-r60 PMC3218848 21702898

[21] Nearing JT, Douglas GM, Hayes MG, MacDonald J, Desai DK, Allward N, et al. Microbiome differential abundance methods produce different results across 38 datasets. Nat Commun. 2022;13:342. 10.1038/s41467-022-28034-z PMC8763921 35039521

[22] Mallick H, Rahnavard A, McIver LJ, Ma S, Zhang Y, Nguyen LH, et al. Multivariable association discovery in population-scale meta-omics studies. PLoS Comput Biol. 2021;17(11):e1009442. 10.1371/journal.pcbi.1009442 PMC8714082 34784344

[23] Antonio MAD, Meyn LA, Murray PJ, Busse B, Hillier SL (2009). Vaginal colonization by probiotic lactobacillus crispatus CTV-05 is decreased by sexual activity and endogenous lactobacilli. J Infect Dis.

[24] Ngugi BM, Hemmerling A, Bukusi EA, Kikuvi G, Gikunju J, Shiboski S (2011). Effects of bacterial vaginosis-associated bacteria and sexual intercourse on vaginal colonization with the probiotic lactobacillus crispatus CTV-05. Sex Transm Dis.

[25] Zmora N, Zilberman-Schapira G, Suez J, Mor U, Dori-Bachash M, Bashiardes S (2018). Personalized gut mucosal colonization resistance to empiric probiotics is associated with unique host and microbiome features. Cell.

[26] Oduyebo OO, Anorlu RI, Ogunsola FT. The effects of antimicrobial therapy on bacterial vaginosis in non-pregnant women. Cochrane Database Syst Rev. 2009;(3):CD006055. 10.1002/14651858.CD006055.pub2 19588379

