# Supplementary material for: Vaginal Lactobacillus crispatus persistence following application of a live biotherapeutic product: colonization phenotypes and genital immune impact
Source: Microbiome. 2024 Jun 21;12:110. doi: 10.1186/s40168-024-01828-7 (PMC11191164; doi:10.1186/s40168-024-01828-7)
Supplement: Supplementary file 1 — Supplementary Material 1. [file 40168_2024_1828_MOESM1_ESM.docx]

**Supplemental Material**

**
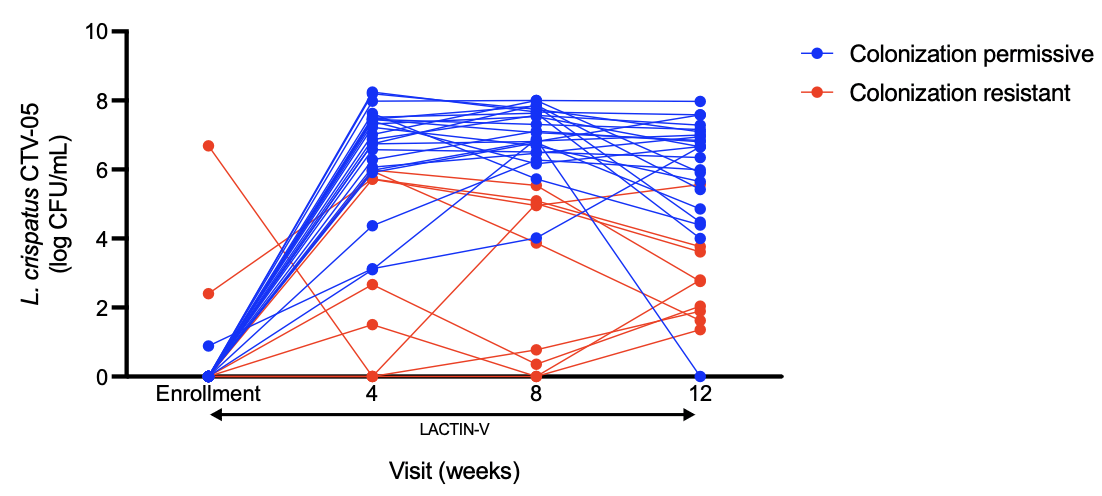
**

**Supplementary figure S1: Longitudinal dynamics of *L. crispatus* CTV-05 among colonization resistant and permissive groups.** Absolute abundance of *L. crispatus* CTV-05 for each participant in the colonization permissive and resistant groups at enrollment and weeks 4, 8, and 12.


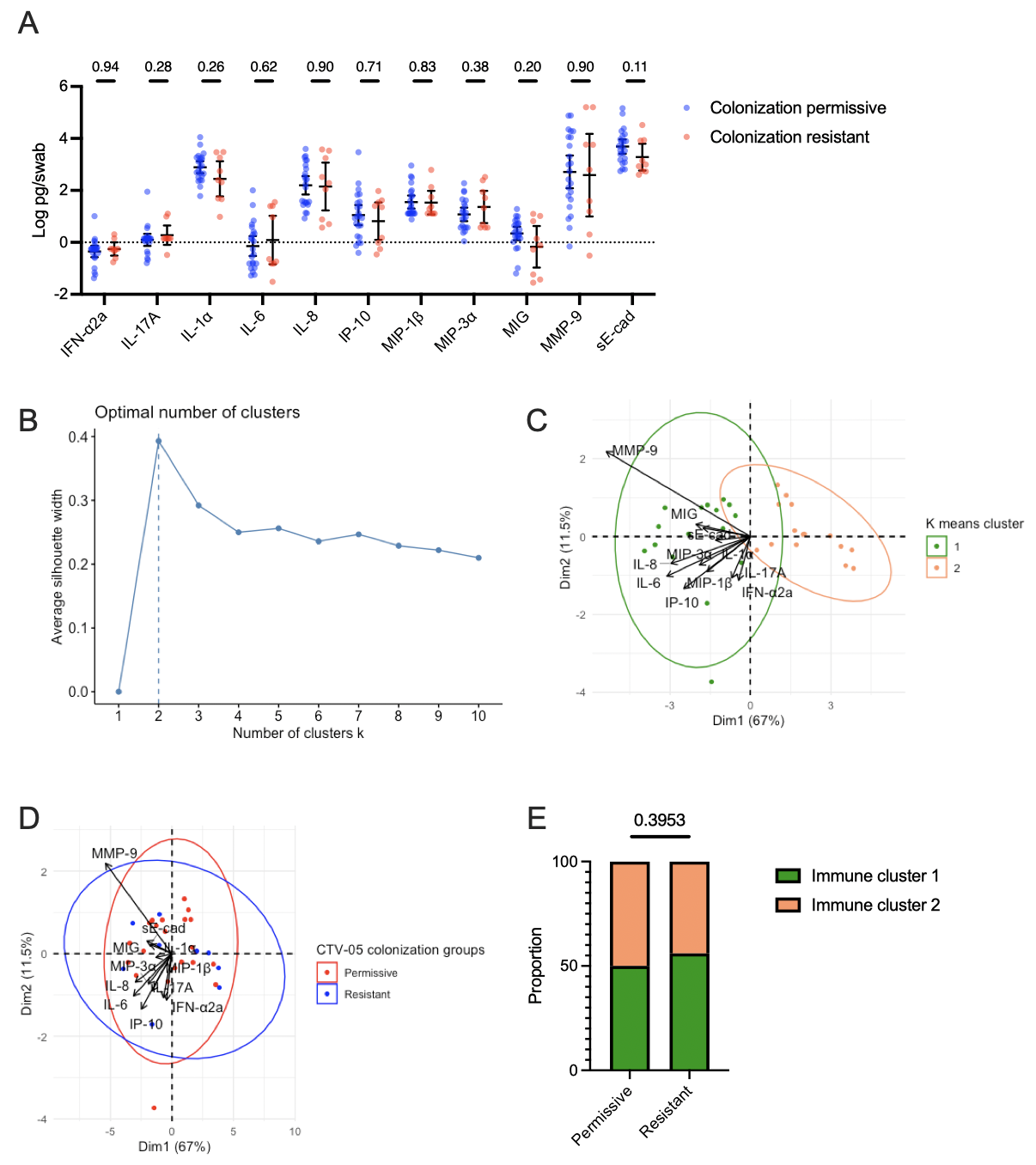


**Supplementary figure S2: Genital immune milieu prior to LACTIN-V administration does not predict colonization resistance.** A) Comparison of genital immune factors prior to LACTIN-V administration between colonization resistant and permissive women (n=32, Mann-Whitney U test). Data points and error bars are mean and 95% confidence intervals, respectively. B) Silhouette analysis results for optimal number of immune clusters by k means clustering using immune data at the pre-LACTIN-V visit for all participants with complete immune data at this visit (n=31). Principal component analysis plots for immune data, coloured according to C) k means clustering groups and D) CTV-05 colonization groups (n=31). E) Comparison of the proportion of colonization resistant and permissive individuals belonging to each pre-LACTIN-V immune cluster identified with k means clustering (n=31, Pearson Chi-Square test).

**
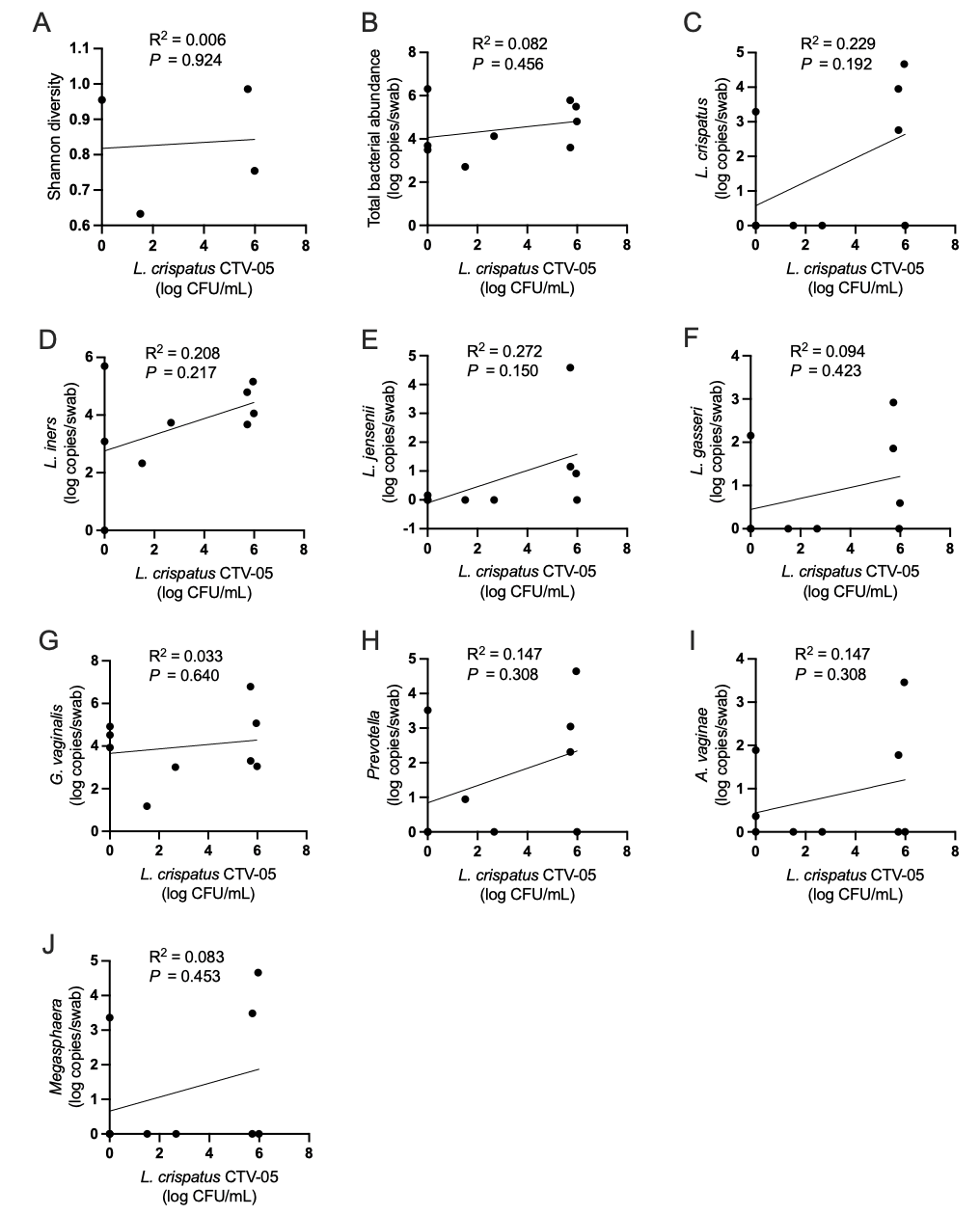
**

**Supplementary figure S3: Vaginal microbiota characteristics prior to LACTIN-V administration do not predict *L. crispatus* CTV-05 absolute abundance in the colonization resistant group.** Association between *L. crispatus* CTV-05 at the first visit after the start of LACTIN-V administration (i.e., the 4-week visit) and A) vaginal microbiota Shannon diversity (n=4), B) total bacterial load, and absolute abundance of C) *L. crispatus*, D) *L. iners*, E) *L. jensenii*, F) *L. gasseri*, G) *G. vaginalis*, H) *Prevotella* spp., I) *A. vaginae*, and J) *Megasphaera* spp. (n=9). R^2^ and *p* values obtained with linear regression.


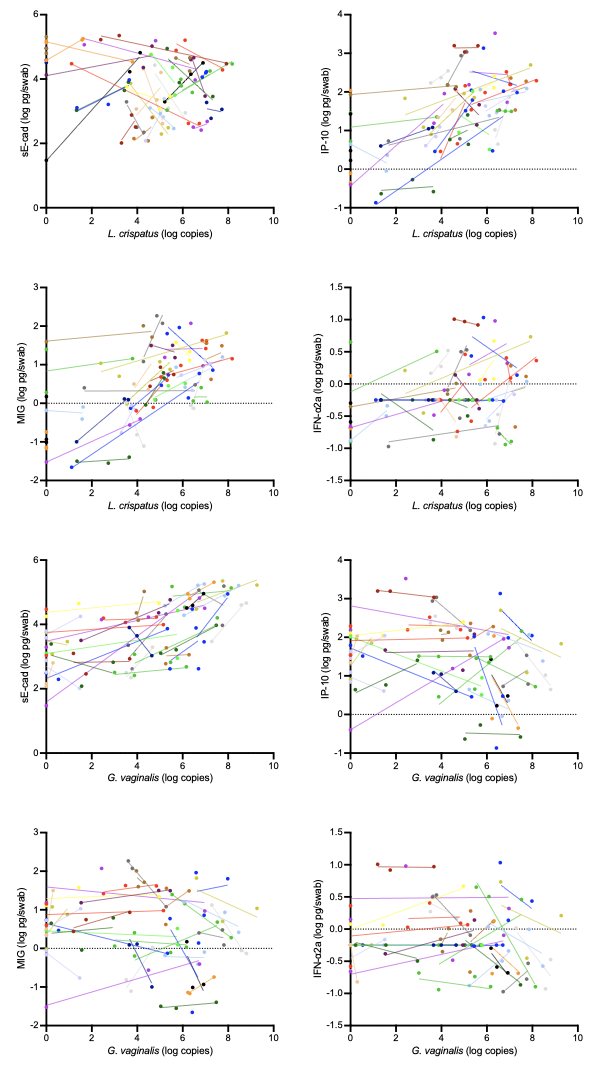


**Supplemental figure S4: Repeated measures associations between immune factors and bacteria linked with *L. crispatus* CTV-05 colonization resistance/permissiveness.** Visualization of the repeated measures associations between vaginal levels of sE-cad, IP-10, MIG, and IFN-α2a and absolute abundance of *L. crispatus* and *G. vaginalis* during LACTIN-V administration. Data points and regression lines coloured according to individual participants. *P* values and linear mixed model coefficients presented in Table 1.


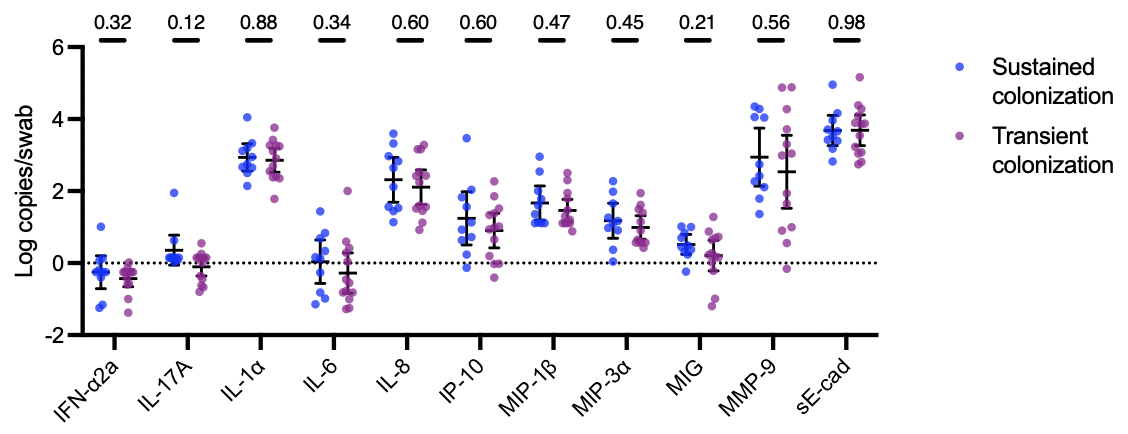


**Supplementary figure S5: Vaginal immune factors prior to LACTIN-V administration do not predict sustained/transient colonization.** Comparison of genital immune factors prior to LACTIN-V administration between sustained and colonization groups (n=23, Mann-Whitney U test). Data points and error bars are mean and 95% confidence intervals, respectively.


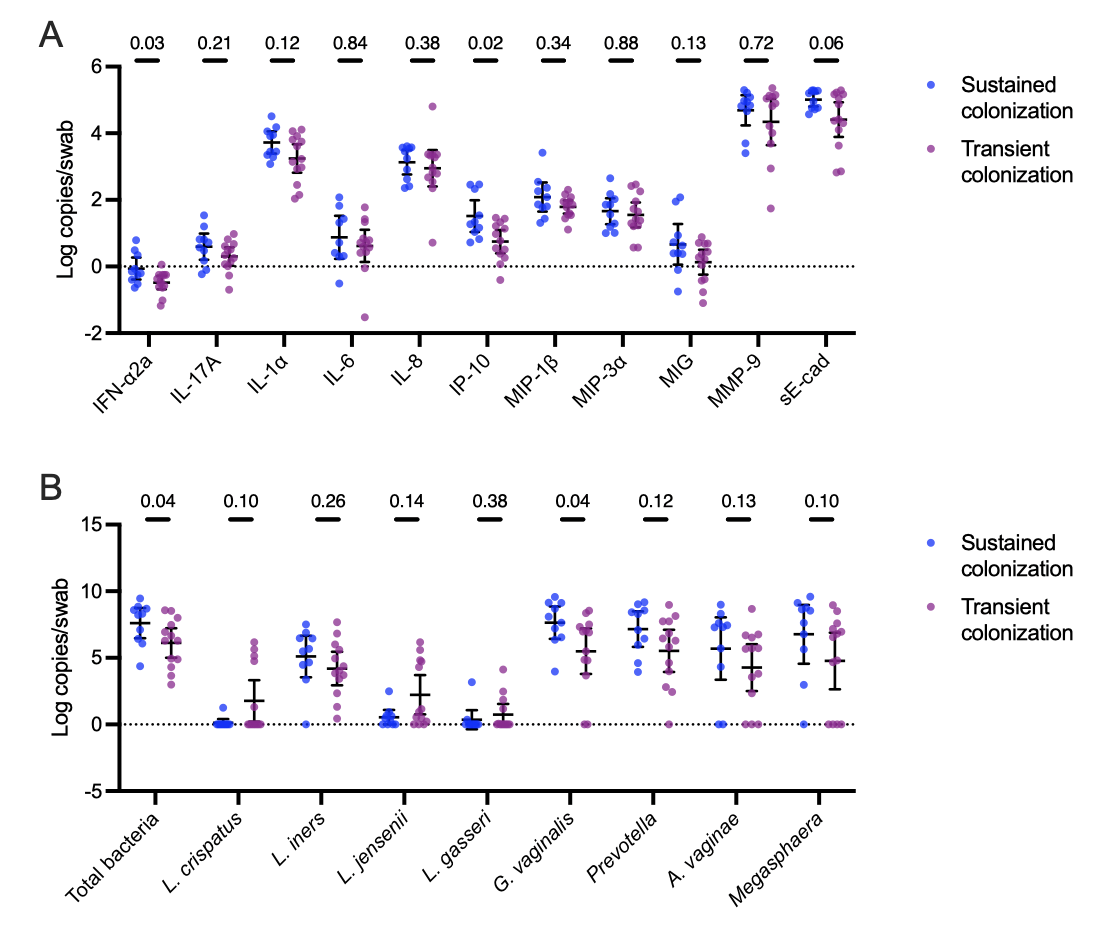


**Supplementary figure S6: Elevated bacterial absolute abundances prior to metronidazole treatment among women with sustained colonization.** Comparison of A) genital immune factors and B) bacterial absolute abundances prior to metronidazole treatment between sustained and colonization groups (n=23, Mann-Whitney U test). Data points and error bars are mean and 95% confidence intervals, respectively.


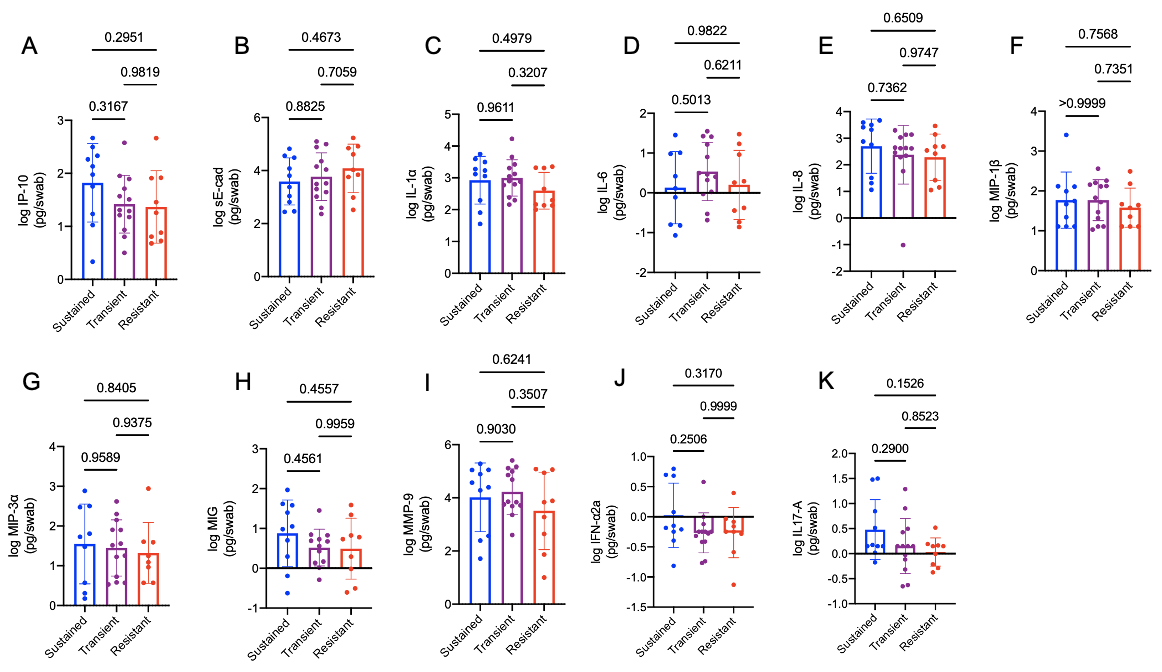


**Supplemental figure S7: Genital immune factors do not differ between colonization groups three months after the last dose of LACTIN-V.** Vaginal levels of A) IP-10, B) sE-cad, C) IL-1α, D) IL-6, E) IL-8, F) MIP-1β, G) MIP-3α, H) MIG, I) MMP-9, J) IFN-α2a, and K) IL-17A at 24 weeks (i.e., three months after the last dose of LACTIN-V). P values for pairwise comparisons performed with the Tukey post-hoc test following ANOVA.


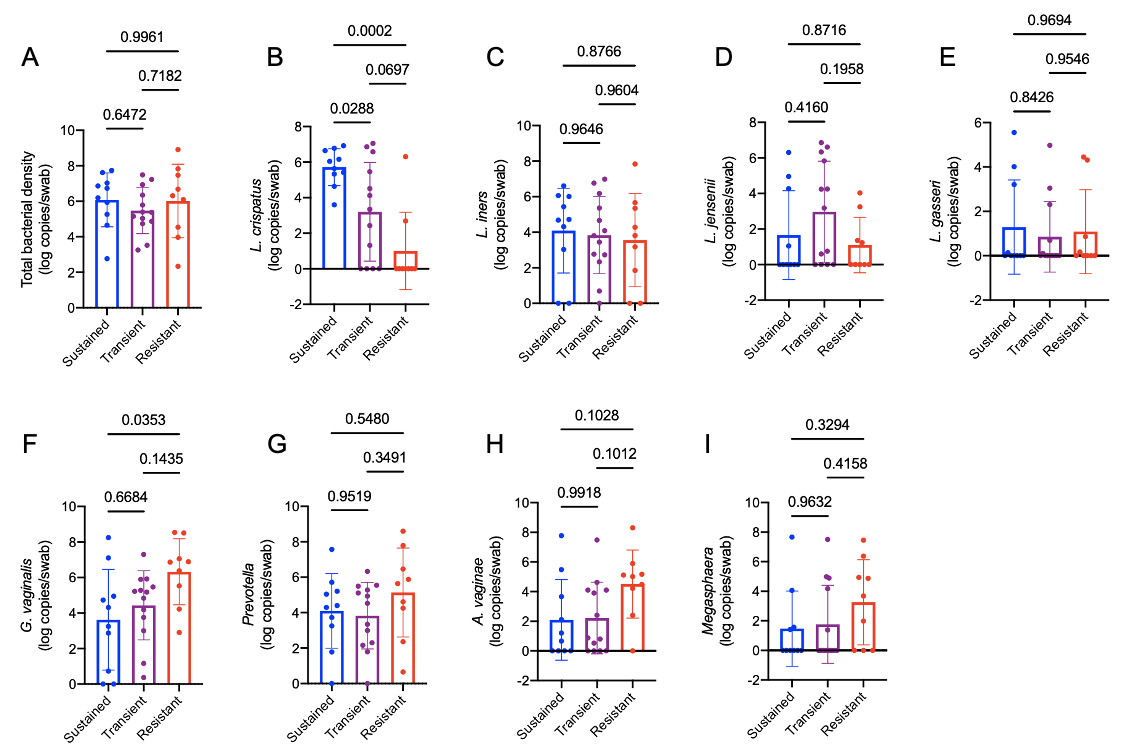


**Supplemental figure S8: Elevated *L. crispatus* and reduced BV-associated bacteria among sustained colonization group three months after the last dose of LACTIN-V.** Vaginal A) total bacterial load and absolute abundance of B) *L. crispatus*, C) *L. iners*, D) *L. jensenii*, E) *L. gasseri*, F) *G. vaginalis*, G) *Prevotella* spp., H) *A. vaginae*, and I) *Megasphaera* spp. at 24 weeks (i.e., three months after the last dose of LACTIN-V). P values for pairwise comparisons performed with the Tukey post-hoc test following ANOVA.

|  | **Participants included in sub-analysis**  **(n=32)** | **Other participants in LACTIN-V arm**  **(n=119)** | **P value** |
| --- | --- | --- | --- |
| **Age** | 31.5 (26.75-36.25) | 29 (25.5-34.0) | 0.2278 |
| **Race – Asian** | 3 (9%) | 3 (3%) | 0.2104 |
| **Race – Black** | 14 (44%) | 49 (41%) | 0.952 |
| **Race – multi-racial** | 0 (0%) | 10 (8%) | 0.1948 |
| **Race – unknown** | 1 (3%) | 13 (11%) | 0.3139 |
| **Race – White** | 14 (44%) | 41 (34%) | 0.4453 |
| **Race – American Indian or Alaska Native** | 0 (0%) | 2 (2%) | 1.0000 |
| **Race – Native Hawaiian or other Pacific Islander** | 0 (0%) | 1 (1%) | 1.0000 |
| **Hormonal contraceptive use** | 7 (22%) | 20 (17%) | 0.6859 |
| **Missed doses of LACTIN-V** | 0.5 (0-2) | 1 (0-4) | 0.293 |
| **Sexual intercourse within 30 days of metronidazole treatment** | 24 (75%) | 98 (82%) | 0.4936 |
| **Sexual intercourse during metronidazole treatment** | 8 (25%) | 39 (33%) | 0.53 |
| **Sexual intercourse during LACTIN-V application** | 26 (81%) | 86 (72%) | 0.9676 |
| **Number of sex acts during LACTIN-V application** | 7 (1.75- 15.25) | 5 (1-13) | 0.6038 |
| **Number of sex acts during and after LACTIN-V application** | 16.5 (4.75-34.75) | 11 (4-24) | 0.3608 |
| **New sex partner during LACTIN-V application** | 8 (25%) | 25 (21%) | 0.996 |
| **New sex partner during or after LACTIN-V application** | 14 (44%) | 41 (34%) | 0.702 |
| Data are median (interquartile range) or frequency (percent).  P values generated with the Mann-Whitney U test for continuous variables and the Pearson Chi-Square test for frequencies of categorical variables. | | | |

**Supplementary table S1: Comparison of sociodemographic characteristics, missed doses, and sexual activity between participants included in the present sub-analysis and all other participants randomized to receive LACTIN-V in the larger clinical trial.**

| **Target** | **Genomic region** | **Oligo** | **Sequence** |
| --- | --- | --- | --- |
| 16S | 16S rRNA | Forward | 5-TCCTACGGGAGGCAGCAGT-3 |
|  |  | Reverse | 5-GGACTACCAGGGTATCTAATCCTGTT-3 |
|  |  | Probe | (FAM)-CGTATTACCGCGGCTGCTGGCAC-(NFQ-MGB) |
| *L. crispatus* | *tuf* | Forward | CGTGGTTCAGCWTTGAAGGC |
|  |  | Reverse | CTTCAACTGGCATYAAGAATGGC |
|  |  | Probe | [ROX]-AGGCGACAAGGAAGCTCAAGAAC-BHQ2 |
| *L. iners* | *tuf* | Forward | CGTGGTTCAGCWTTGAAGGC |
|  |  | Reverse | CTTCAACTGGCATYAAGAATGGC |
|  |  | Probe | [HEX]-AGGCGATCCAGAACAAGAAGCAG-BHQ1 |
| *L. gasseri* | *tuf* | Forward | CGTGGTTCAGCWTTGAAGGC |
|  |  | Reverse | CTTCAACTGGCATYAAGAATGGC |
|  |  | Probe | [FAM]-AGGTGACCCAGAACAACAAGACG-BHQ1 |
| *L. jensenii* | *tuf* | Forward | CGTGGTTCAGCWTTGAAGGC |
|  |  | Reverse | CTTCAACTGGCATYAAGAATGGC |
|  |  | Probe | [Cy5]-AGGTGACCCAGAACAAGAAAAGGT-BHQ2 |
| *G. vaginalis* | 16S rRNA | Forward | GCGGGCTAGAGTGCA |
|  |  | Reverse | ACCCGTGGAATGGGCC |
|  |  | Probe | [ROX]CTTCTCAGCGTCAGTAACAGC |
| *A. vaginae* | 16S rRNA | Forward | TAGGTCAGGAGTTAAATCTG |
|  |  | Reverse | TCATGGCCCAGAAGACCGCC |
|  |  | Probe | [HEX]CTACCAGACTCAAGCCTGCC |
| *Megasphaera* | 16S rRNA | Forward | GATGCCAACAGTATCCGTCCG |
|  |  | Reverse | CCTCTCCGACACTCAAGTTCGA |
|  |  | Probe | [FAM]ACAGACTTACCGAACCGCCT |
| *Prevotella* | 16S rRNA | Forward | 5-CCAGCCAAGTAGCGTGCA-3 |
|  |  | Reverse | 5-TGGACCTTCCGTATTACCGC-3 |
|  |  | Probe | (56-FAM)-AATAAGGACCGGCTAATTCCGTGCCAG-(36-TAMSp) |

**Supplementary table S2.** **Primer and probe sequences for quantitative polymerase chain reaction assays.**

| **Target** | **Oligo** | **Sequence** |
| --- | --- | --- |
| *L. crispatus* | Forward | AAAGTCCTGGTTTGATCTGCGT |
|  | Reverse | CACTTCCTAGCCACTGTGTTGT |
| *L. crispatus* CTV-05 | Forward | GCTGTTGCAGCCAGACAGTT |
|  | Reverse | TCTCTGGGACATCCATAAGTTG |

**Supplementary table S3.** **Primer sequences for *L. crispatus* and CTV-05 polymerase chain reaction assays.**

|  | **Colonization permissive**  **(n=23)** | **Colonization resistant**  **(n=9)** | **P value** |
| --- | --- | --- | --- |
| **Age** | 30 (26.5-35) | 33 (27-44) | 0.3031 |
| **Race – Asian** | 13% | 0% | 0.6429 |
| **Race – Black** | 35% | 67% | 0.2156 |
| **Race – unknown** | 0% | 11% | 0.6211 |
| **Race – White** | 52% | 22% | 0.2546 |
| **Hormonal contraceptive use** | 22% | 29% | 1.0000 |
| **Missed doses of LACTIN-V** | 0 (0-1.5) | 1 (0-3) | 0.4441 |
| **Sexual intercourse within 30 days of metronidazole treatment** | 74% | 78% | 1.0000 |
| **Sexual intercourse during metronidazole treatment** | 22% | 33% | 0.8204 |
| **Sexual intercourse during LACTIN-V application** | 78% | 89% | 0.8502 |
| **Number of sex acts during LACTIN-V application** | 6 (1-15.5) | 10 (5-13) | 0.5415 |
| **Number of sex acts during and after LACTIN-V application** | 20 (4.5-31) | 16 (9-38) | 0.7848 |
| **New sex partner during LACTIN-V application** | 22% | 33% | 0.8204 |
| **New sex partner during or after LACTIN-V application** | 48% | 33% | 0.7288 |
| Data are median (interquartile range) or frequency.  P values generated with the Mann-Whitney U test for continuous variables and the Pearson Chi-Square test for frequencies of categorical variables. | | | |

**Supplementary table S4: Comparison of sociodemographic characteristics, missed doses, and sexual activity between *L. crispatus* CTV-05 colonization resistant and permissive women.**

| **Species** | **Log average of highest class** | **Highest class** | **LDA effect size** | **P value** | **FDR adjusted P value** |
| --- | --- | --- | --- | --- | --- |
| *Peptoniphilus duerdenii* | 2.01 | Resistant | 1.68 | 0.01 | 0.64 |
| *Staphylococcus epidermidis* | 2.37 | Resistant | 2.12 | 0.06 | 0.64 |
| *Corynebacterium jeikeium* | 0.87 | Resistant | 3.72 | 0.10 | 0.64 |
| *Streptococcus constellatus* | 1.27 | Resistant | 3.18 | 0.10 | 0.64 |
| *Klebsiella oxytoca* | 1.11 | Resistant | 3.14 | 0.10 | 0.64 |
| *Streptococcus sanguinis* | 0.81 | Resistant | 2.82 | 0.10 | 0.64 |
| *Actinomyces europaeus* | 1.90 | Resistant | 2.71 | 0.10 | 0.64 |
| *Brevibacterium mcbrellneri* | 1.90 | Resistant | 2.65 | 0.10 | 0.64 |
| *Porphyromonas asaccharolytica* | 2.37 | Resistant | 2.63 | 0.10 | 0.64 |
| *Streptococcus infantarius* | 1.86 | Resistant | 2.55 | 0.10 | 0.64 |
| *Gemella haemolysans* | 2.16 | Resistant | 2.53 | 0.10 | 0.64 |
| *Anaerococcus hydrogenalis* | 1.86 | Resistant | 2.44 | 0.10 | 0.64 |
| *Actinobaculum massiliae* | 2.29 | Resistant | 2.28 | 0.10 | 0.64 |
| *Ureaplasma urealyticum* | 3.09 | Resistant | 2.77 | 0.12 | 0.64 |
| *Peptoniphilus lacrimalis* | 1.72 | Permissive | 1.36 | 0.13 | 0.64 |
| *Propionibacterium* sp | 2.89 | Resistant | 2.54 | 0.13 | 0.64 |
| *Prevotella corporis* | 2.15 | Resistant | 2.32 | 0.13 | 0.64 |
| *Streptococcus anginosus* | 2.08 | Resistant | 1.72 | 0.13 | 0.64 |
| *Megasphaera genomosp* | 3.23 | Permissive | 2.89 | 0.18 | 0.65 |
| *Dialister micraerophilus* | 2.07 | Permissive | 1.77 | 0.18 | 0.65 |
| *Lactobacillus iners* | 5.78 | Permissive | 5.03 | 0.19 | 0.65 |
| *Clostridium* sp | 1.95 | Permissive | 2.54 | 0.26 | 0.65 |
| *Dialister microaerophilus* | 1.51 | Permissive | 1.86 | 0.26 | 0.65 |
| *Prevotella buccalis* | 2.06 | Permissive | 1.73 | 0.26 | 0.65 |
| *Megasphaera* sp | 1.85 | Permissive | 1.54 | 0.26 | 0.65 |
| *Facklamia hominis* | 1.25 | Permissive | 1.03 | 0.26 | 0.65 |
| *Megasphaera micronuciformis* | 1.24 | Permissive | 0.97 | 0.26 | 0.65 |
| *Finegoldia magna* | 2.33 | Resistant | 1.98 | 0.28 | 0.65 |
| *Prevotella amnii* | 3.03 | Permissive | 2.71 | 0.30 | 0.65 |
| *Prevotella timonensis* | 2.29 | Permissive | 1.98 | 0.30 | 0.65 |
| *Lactobacillus johnsonii* | 1.73 | Resistant | 1.26 | 0.35 | 0.65 |
| *Lactobacillus gasseri* | 4.18 | Resistant | 3.88 | 0.35 | 0.65 |
| *Prevotella* sp | 1.37 | Permissive | 1.01 | 0.38 | 0.65 |
| *Escherichia coli* | 3.74 | Resistant | 3.72 | 0.38 | 0.65 |
| *Streptococcus salivarius* | 0.81 | Resistant | 3.17 | 0.38 | 0.65 |
| *Staphylococcus haemolyticus* | 1.28 | Resistant | 3.05 | 0.38 | 0.65 |
| *Streptococcus urinalis* | 1.12 | Permissive | 2.91 | 0.38 | 0.65 |
| *Lactobacillus fermentum* | 0.87 | Resistant | 2.83 | 0.38 | 0.65 |
| *Actinomyces neuii* | 1.13 | Permissive | 2.42 | 0.38 | 0.65 |
| *Arcanobacterium* sp | 1.32 | Permissive | 2.32 | 0.38 | 0.65 |
| *Fusobacterium gonidiaformans* | 1.71 | Permissive | 2.28 | 0.38 | 0.65 |
| *Prevotella pallens* | 1.07 | Permissive | 2.27 | 0.38 | 0.65 |
| *Corynebacterium amycolatum* | 1.90 | Resistant | 2.26 | 0.38 | 0.65 |
| *Streptococcus dysgalactiae* | 0.57 | Permissive | 2.15 | 0.38 | 0.65 |
| *Peptostreptococcus anaerobius* | 1.21 | Permissive | 2.07 | 0.38 | 0.65 |
| *Ruminococcus lactaris* | 1.96 | Permissive | 2.06 | 0.38 | 0.65 |
| *Lactobacillus kefiranofaciens* | 2.15 | Permissive | 1.88 | 0.38 | 0.65 |
| *Corynebacterium pseudogenitalium* | 0.99 | Permissive | 1.82 | 0.38 | 0.65 |
| *Peptostreptococcus* sp | 0.77 | Permissive | 1.74 | 0.38 | 0.65 |
| *Prevotella bergensis* | 0.93 | Permissive | 1.59 | 0.38 | 0.65 |
| *Filifactor alocis* | 1.01 | Permissive | 1.29 | 0.38 | 0.65 |
| *Gardnerella vaginalis* | 5.39 | Resistant | 4.97 | 0.43 | 0.66 |
| *Enterococcus faecium* | 1.11 | Resistant | 0.85 | 0.51 | 0.66 |
| *Streptococcus bovis* | 1.35 | Permissive | 3.52 | 0.55 | 0.66 |
| *Anaerococcus vaginalis* | 1.22 | Permissive | 3.47 | 0.55 | 0.66 |
| *Veillonella atypica* | 1.15 | Permissive | 3.40 | 0.55 | 0.66 |
| *Lactobacillus amylovorus* | 1.57 | Permissive | 3.35 | 0.55 | 0.66 |
| *Streptococcus mitis* | 0.42 | Permissive | 3.34 | 0.55 | 0.66 |
| *Lactobacillus helveticus* | 0.55 | Permissive | 3.08 | 0.55 | 0.66 |
| *Anaerococcus lactolyticus* | 1.03 | Permissive | 2.90 | 0.55 | 0.66 |
| *Treponema phagedenis* | 0.29 | Permissive | 2.86 | 0.55 | 0.66 |
| *Porphyromonas uenonis* | 0.69 | Permissive | 2.78 | 0.55 | 0.66 |
| *Porphyromonas somerae* | 0.42 | Permissive | 2.74 | 0.55 | 0.66 |
| *Streptococcus parasanguinis* | 0.25 | Permissive | 2.74 | 0.55 | 0.66 |
| *Mycoplasma genitalium* | 0.42 | Permissive | 2.60 | 0.55 | 0.66 |
| *Prevotella tannerae* | 0.60 | Permissive | 2.58 | 0.55 | 0.66 |
| *Prevotella stercorea* | 0.46 | Permissive | 2.55 | 0.55 | 0.66 |
| *Prevotella melaninogenica* | 0.51 | Permissive | 2.53 | 0.55 | 0.66 |
| *Peptoniphilus asaccharolyticus* | 1.13 | Permissive | 2.37 | 0.55 | 0.66 |
| *Bifidobacterium longum* | 0.51 | Permissive | 2.35 | 0.55 | 0.66 |
| *Prevotella buccae* | 0.60 | Permissive | 2.14 | 0.55 | 0.66 |
| BVAB3 | 2.23 | Permissive | 1.89 | 0.56 | 0.66 |
| *Prevotella bivia* | 1.92 | Permissive | 1.51 | 0.56 | 0.66 |
| *Atopobium vaginae* | 3.07 | Permissive | 2.34 | 0.56 | 0.66 |
| *Ureaplasma parvum* | 2.70 | Resistant | 2.29 | 0.57 | 0.67 |
| *Prevotella disiens* | 1.27 | Permissive | 0.96 | 0.59 | 0.68 |
| *Anaerococcus tetradius* | 2.03 | Resistant | 1.69 | 0.64 | 0.73 |
| *Bifidobacterium breve* | 3.91 | Permissive | 3.58 | 0.66 | 0.73 |
| *Lactobacillus coleohominis* | 2.81 | Resistant | 2.47 | 0.66 | 0.73 |
| *Lactobacillus crispatus* | 4.93 | Resistant | 4.57 | 0.70 | 0.76 |
| *Streptococcus agalactiae* | 4.14 | Permissive | 3.82 | 0.70 | 0.76 |
| *Mobiluncus mulieris* | 2.49 | Permissive | 2.18 | 0.74 | 0.79 |
| *Enterococcus faecalis* | 4.83 | Resistant | 4.59 | 0.77 | 0.81 |
| *Mycoplasma hominis* | 2.36 | Permissive | 2.52 | 0.78 | 0.82 |
| *Lactobacillus jensenii* | 5.43 | Permissive | 4.99 | 0.79 | 0.82 |
| *Peptoniphilus harei* | 2.37 | Resistant | 2.08 | 0.87 | 0.88 |
| *Clostridiales* Family | 1.24 | Permissive | 0.91 | 0.87 | 0.88 |
| BVAB1 | 3.33 | Permissive | 2.56 | 0.95 | 0.95 |

**Supplemental table S5: Linear discriminant analysis (LDA) effect size (LEfSe) results comparing the relative abundance of all bacterial species immediately after metronidazole treatment between colonization resistant and permissive participants.**

|  | **Sustained colonization**  **(n=10)** | **Transient colonization**  **(n=13)** | **P value** |
| --- | --- | --- | --- |
| **Age** | 30 (27.75-32.50) | 33 (26-36) | 0.4541 |
| **Race – Asian** | 20% | 8% | 0.807 |
| **Race – Black** | 20% | 46% | 0.3876 |
| **Race – unknown** | 0% | 0% | NA |
| **Race – White** | 60% | 46% | 0.8119 |
| **Hormonal contraceptive use** | 20% | 23% | 1 |
| **Missed doses of LACTIN-V** | 0.5 (0-1.75) | 0 (0-1) | 0.7361 |
| **Sexual intercourse within 30 days of metronidazole treatment** | 80% | 69% | 0.9171 |
| **Sexual intercourse during metronidazole treatment** | 30% | 15% | 0.7395 |
| **Sexual intercourse during LACTIN-V application** | 80% | 77% | 1 |
| **Number of sex acts during LACTIN-V application** | 5 (1.25-12.50) | 6 (1-18) | 0.6849 |
| **Number of sex acts during and after LACTIN-V application** | 23 (7.25-32.00) | 17 (4-28) | 0.8036 |
| **New sex partner during LACTIN-V application** | 40% | 8% | 0.1763 |
| **New sex partner after LACTIN-V application** | 40% | 46% | 1 |
| Data are median (interquartile range) or frequency.  P values generated with the Mann-Whitney U test for continuous variables and the Pearson Chi-square test for frequencies of categorical variables. | | | |

**Table S10: Comparison of sociodemographic characteristics, missed doses, and sexual activity between sustained and transient colonization groups.**

| **Species** | **Log of highest class average** | **Highest class** | **LDA effect size** | **P value** | **FDR adjusted p value** |
| --- | --- | --- | --- | --- | --- |
| *Clostridium* sp | 2.291961484 | Sustained | 1.983990634 | 0.03662753 | 0.722621 |
| *Facklamia hominis* | 1.510534132 | Transient | 1.249254946 | 0.0815826 | 0.722621 |
| *Lactobacillus jensenii* | 5.562108975 | Transient | 4.993999339 | 0.10034825 | 0.722621 |
| *Atopobium vaginae* | 3.19389201 | Sustained | 2.482950726 | 0.10034825 | 0.722621 |
| *Prevotella pallens* | 1.410471449 | Sustained | 2.074842515 | 0.10420354 | 0.722621 |
| *Peptostreptococcus anaerobius* | 1.554085786 | Sustained | 1.692849628 | 0.10420354 | 0.722621 |
| *Actinomyces neuii* | 1.469349797 | Sustained | 1.261802247 | 0.10420354 | 0.722621 |
| *Streptococcus anginosus* | 1.635850084 | Transient | 2.061878536 | 0.17573434 | 0.722621 |
| *Corynebacterium pseudogenitalium* | 1.250715199 | Transient | 1.851445599 | 0.17573434 | 0.722621 |
| *Streptococcus urinalis* | 1.382406476 | Transient | 1.327974679 | 0.17573434 | 0.722621 |
| *Gardnerella vaginalis* | 4.972557446 | Sustained | 4.383142178 | 0.20124262 | 0.722621 |
| *Bifidobacterium breve* | 4.167593819 | Transient | 3.991231104 | 0.20452609 | 0.722621 |
| *Lactobacillus iners* | 5.863319796 | Sustained | 5.029606736 | 0.27332168 | 0.722621 |
| *Mycoplasma genitalium* | 0.765881449 | Sustained | 2.600103157 | 0.27332168 | 0.722621 |
| *Prevotella buccae* | 0.941972212 | Sustained | 2.573461692 | 0.27332168 | 0.722621 |
| *Porphyromonas somerae* | 0.765881449 | Sustained | 2.535729491 | 0.27332168 | 0.722621 |
| *Staphylococcus epidermidis* | 1.422937756 | Sustained | 2.502282624 | 0.27332168 | 0.722621 |
| *Staphylococcus haemolyticus* | 0.997801198 | Sustained | 2.384140769 | 0.27332168 | 0.722621 |
| *Escherichia coli* | 1.820879076 | Sustained | 2.196658836 | 0.27332168 | 0.722621 |
| *Prevotella tannerae* | 0.941972212 | Sustained | 2.177442235 | 0.27332168 | 0.722621 |
| *Veillonella atypica* | 1.49400969 | Sustained | 1.435878229 | 0.27332168 | 0.722621 |
| *Streptococcus mitis* | 0.767011704 | Sustained | 1.336774271 | 0.27332168 | 0.722621 |
| *Lactobacillus helveticus* | 0.891950812 | Sustained | 1.162155297 | 0.27332168 | 0.722621 |
| *Streptococcus salivarius* | 0.590921931 | Sustained | 0.922174979 | 0.27332168 | 0.722621 |
| *Streptococcus parasanguinis* | 0.590921931 | Sustained | 0.823336518 | 0.27332168 | 0.722621 |
| *Streptococcus agalactiae* | 4.370457322 | Transient | 4.034459894 | 0.29037407 | 0.722621 |
| *Mobiluncus mulieris* | 2.82452547 | Sustained | 2.520783293 | 0.29604801 | 0.722621 |
| *Ureaplasma parvum* | 2.765361629 | Sustained | 2.465402063 | 0.31950029 | 0.722621 |
| *Prevotella timonensis* | 2.503293401 | Sustained | 1.992520465 | 0.31950029 | 0.722621 |
| *Ureaplasma urealyticum* | 2.625707648 | Sustained | 2.296309742 | 0.33835172 | 0.722621 |
| *Corynebacterium amycolatum* | 0.73557347 | Transient | 2.759490967 | 0.36131043 | 0.722621 |
| *Prevotella stercorea* | 0.7270774 | Transient | 2.747935263 | 0.36131043 | 0.722621 |
| *Peptoniphilus duerdenii* | 0.852016137 | Transient | 2.590259925 | 0.36131043 | 0.722621 |
| *Lactobacillus amylovorus* | 1.833747197 | Transient | 2.547699832 | 0.36131043 | 0.722621 |
| *Peptoniphilus asaccharolyticus* | 1.396083018 | Transient | 2.541497884 | 0.36131043 | 0.722621 |
| *Treponema phagedenis* | 0.550986141 | Transient | 2.538194566 | 0.36131043 | 0.722621 |
| *Lactobacillus fermentum* | 0.550986141 | Transient | 2.427248567 | 0.36131043 | 0.722621 |
| *Porphyromonas uenonis* | 0.94892615 | Transient | 2.394909117 | 0.36131043 | 0.722621 |
| *Anaerococcus lactolyticus* | 1.291350311 | Transient | 2.23846204 | 0.36131043 | 0.722621 |
| *Anaerococcus vaginalis* | 1.487741285 | Transient | 1.981465483 | 0.36131043 | 0.722621 |
| *Enterococcus faecium* | 1.17579856 | Transient | 1.877126571 | 0.36131043 | 0.722621 |
| *Bifidobacterium longum* | 0.777859034 | Transient | 1.846523357 | 0.36131043 | 0.722621 |
| *Streptococcus bovis* | 1.612681199 | Transient | 1.6345666 | 0.36131043 | 0.722621 |
| *Prevotella melaninogenica* | 0.777859034 | Transient | 1.632425251 | 0.36131043 | 0.722621 |
| *Megasphaera* sp | 2.146596946 | Sustained | 1.831031913 | 0.41636964 | 0.796533 |
| *Megasphaera micronuciformis* | 1.410471449 | Sustained | 1.018708472 | 0.41636964 | 0.796533 |
| BVAB1 | 3.409836533 | Sustained | 2.535770532 | 0.46520882 | 0.871029 |
| *Megasphaera genomosp* | 3.56256988 | Sustained | 3.220976414 | 0.52583232 | 0.925465 |
| *Lactobacillus coleohominis* | 2.021960436 | Transient | 1.554938986 | 0.52583232 | 0.925465 |
| *Prevotella disiens* | 1.309949281 | Sustained | 0.984956371 | 0.52583232 | 0.925465 |
| *Enterococcus faecalis* | 2.117230267 | Sustained | 1.751845421 | 0.55031299 | 0.94956 |
| *Prevotella bivia* | 2.044461719 | Transient | 1.60182975 | 0.61866544 | 1 |
| *Prevotella buccalis* | 2.272479159 | Transient | 2.03444579 | 0.6423466 | 1 |
| *Lactobacillus johnsonii* | 1.591117121 | Transient | 1.290694251 | 0.6423466 | 1 |
| *Clostridiales* Family | 1.38443184 | Transient | 1.047037182 | 0.6423466 | 1 |
| *Lactobacillus gasseri* | 2.792526281 | Sustained | 2.441546868 | 0.70859681 | 1 |
| *Lactobacillus crispatus* | 4.757033344 | Transient | 4.225518878 | 0.71500065 | 1 |
| *Prevotella amnii* | 3.30619498 | Sustained | 2.914221209 | 0.76521048 | 1 |
| BVAB3 | 2.235760185 | Transient | 1.819232004 | 0.76521048 | 1 |
| *Anaerococcus tetradius* | 1.912009272 | Sustained | 2.232212962 | 0.78654388 | 1 |
| *Ruminococcus lactaris* | 2.066911445 | Sustained | 2.051556683 | 0.78654388 | 1 |
| *Lactobacillus kefiranofaciens* | 2.244133454 | Sustained | 1.902083939 | 0.78654388 | 1 |
| *Filifactor alocis* | 1.066911445 | Sustained | 1.815998174 | 0.78654388 | 1 |
| *Arcanobacterium* sp | 1.457984968 | Sustained | 1.489093172 | 0.78654388 | 1 |
| *Peptoniphilus harei* | 1.74360529 | Sustained | 2.318253208 | 0.81637477 | 1 |
| *Dialister microaerophilus* | 1.651014197 | Sustained | 1.845191417 | 0.81637477 | 1 |
| *Finegoldia magna* | 1.464849965 | Sustained | 1.606464541 | 0.81637477 | 1 |
| *Prevotella* sp | 1.476888399 | Sustained | 0.944675185 | 0.92070276 | 1 |

**Supplemental table S11: Linear discriminant analysis (LDA) effect size (LEfSe) results comparing the relative abundance of all bacterial species immediately after metronidazole treatment between sustained and transient colonization groups.**
